# Experimental evolution of cellular miniaturization reveals a putative mechanism for cell size evolution

**DOI:** 10.1073/pnas.2531280123

**Published:** 2026-06-12

**Authors:** Ana Garoña, Morgane V. Lemos, Andrea Giometto, Marco Fumasoni

**Affiliations:** ^a^ Gulbenkian Institute for Molecular Medicine, Oeiras 2780-156, Portugal; ^b^ School of Civil and Environmental Engineering, Cornell University, Ithaca, NY 14853; ^c^ Faculdade de Medicina da Universidade de Lisboa, Lisbon 1649-028, Portugal

**Keywords:** experimental evolution, evolutionary cell biology, cell size, cell cycle, *S. cerevisiae*

## Abstract

Cell volume, a key determinant of physiology, is maintained by cell size homeostasis. Large deviations from typical size are often harmful, yet cell sizes have diverged drastically during evolution. How size homeostasis evolves to support such diversity without impairing cellular function remains unclear. To address this question, we used experimental evolution to progressively select for smaller *Saccharomyces cerevisiae* cells. Over 1,500 generations, we achieved a fourfold modal volume reduction compared to wild type. Despite this reduction, evolved populations maintained robust size homeostasis and relatively high fitness, revealing that substantial decreases in cell volume can occur without severe growth defects. Whole-genome sequencing and genetic perturbations identified mutations in the G1 cyclin Cln3 and the Target of Rapamycin (TOR) pathway effector Sch9 as major contributors to cell size reduction, with the Greatwall kinase Rim15 signaling cascade further modulating this phenotype. Manipulating these pathways produced a sixfold range in cell size without changes in ploidy, with engineered mutations recapitulating the volumes of evolved lines, and loss-of-function mutations yielding enlarged cells. Our results demonstrate the evolutionary plasticity of cell size homeostasis and reveal a putative mechanism for eukaryotic cell size evolution.

## Significance

Cell size influences nearly every aspect of cellular physiology and varies drastically among eukaryotes, yet how such diversity evolves without compromising cellular function remains unclear. Using experimental evolution in budding yeast, we selected for progressively smaller cells and identified mutations in conserved nutrient-sensing and cell-cycle pathways that enable sustained reductions in cell size with minimal fitness cost. These findings show that gradual tuning of core regulatory networks can decouple strong decreases in cell size from growth defects, providing an experimental framework to study the evolution of fundamental cellular traits.

Cells are the fundamental unit of life, and their size is a key determinant of cell and organismal physiology (1). In microbial systems, variation in cell size can shape nutrient acquisition, metabolic scaling, and growth rate, making it a key target of evolutionary and ecological pressures (2). Through its effects on biosynthesis and proliferation, cell size also constrains tissue architecture and regulates functional specialization (3–5).

Not surprisingly, cell size varies greatly across the tree of life. Among prokaryotes, cell diameters range from ∼0.2 μm in *Mycoplasma* species (6) to nearly 1 cm for *Thiomargarita magnifica* (7). Eukaryotes exhibit a similarly broad distribution, ranging from the smallest known free-living species *Ostreococcus tauri,* with a 1 to 2 μm diameter (8), to 15 cm long ostrich eggs (9). Even within multicellular organisms, such as humans, cells range from micron-scale lymphocytes to meter-long neurons (10).

Despite this large variability, specific cell types typically maintain their size within a narrow range through a process known as cell size homeostasis (11, 12). At its core, cell size homeostasis relies on the precise coordination of cell growth and division. The most direct evidence of such control on cell size is that cells born small typically grow more before dividing, and vice versa (13). While size control could in theory be performed throughout the cell cycle (14), most organisms exert it mainly prior to DNA synthesis, such as budding yeast (15) and mammals (16), or before nuclear division, like fission yeast (17). The general principles followed by cells to regulate their size, as well as the molecular mechanisms that execute this control, have been studied and debated for decades. Cell size control is best understood in the budding yeast *Saccharomyces cerevisiae*, where cells are thought to commit to division once they reach a critical size (15) or after adding a constant volume each cycle (18). The mechanism that determines the transition from G1 to S phase has been proposed to depend mainly on the titration of cell cycle activators (e.g., Cln3), inhibitors (e.g., Whi5), transcription factors (e.g., SBF), or their combination (for detailed mechanisms, see refs. 19–26).

Strong evidence for the importance of cell size homeostasis is represented by the severe defects experienced by cells as they leave their narrow range of stereotypical sizes. Excessive cell size has been shown to cause cytoplasm dilution and genetic instability, increasing cells’ propensity to senescence (27–34). On the other hand, mutations causing the largest decrease in cell size impair the Target of Rapamycin (TOR) signaling pathway and ribosome biogenesis, imposing severe reductions in cellular biosynthesis and fitness (35–38). Furthermore, altered cell size control has been observed in cancers (39–42).

It is therefore unclear how evolution achieved changes in cell size spanning many orders of magnitude, when even slight variations are associated with severe cellular defects. These detrimental consequences could severely constrain the evolution of the cell size trait, posing an apparent paradox between the broad evolutionary diversity of cell sizes and the limited tolerance observed within species. To address this paradox, we sought to observe in real time the evolution of a large decrease in cell size using the model eukaryote *S. cerevisiae.* To this goal, we resorted to an experimental evolution regime (43–45), simultaneously imposing direct selection on cellular size by fluorescence-activated cell sorting (FACS), followed by selection for competitive fitness. Applying this regime over 1,500 generations produced miniaturized cells with modal volumes reduced by fourfold relative to wild type (WT), with only marginal fitness tradeoffs. Miniaturized cells maintained their phenotype in the absence of size-based selection and had size homeostasis comparable to their ancestor. Whole-genome sequencing revealed adaptive mutations in G1 cyclins and TOR pathways, with additional contributions from components of the Greatwall kinase signaling cascade. Reconstruction of these mutations showed that their combined effects generate markedly smaller cells while maintaining a near WT fitness, thereby decoupling cell size from growth rate. Targeted engineering of these pathways substantially altered cell size control and cell cycle dynamics, yielding a sixfold range in cell volume. Our work demonstrates the plasticity of the mechanisms governing cell size homeostasis and suggests a putative mechanism for cell size evolution.

## Results

### Experimental Evolution Under Size Selection Produces Cellular Miniaturization in *S. cerevisiae*.

**Fig. 1.**
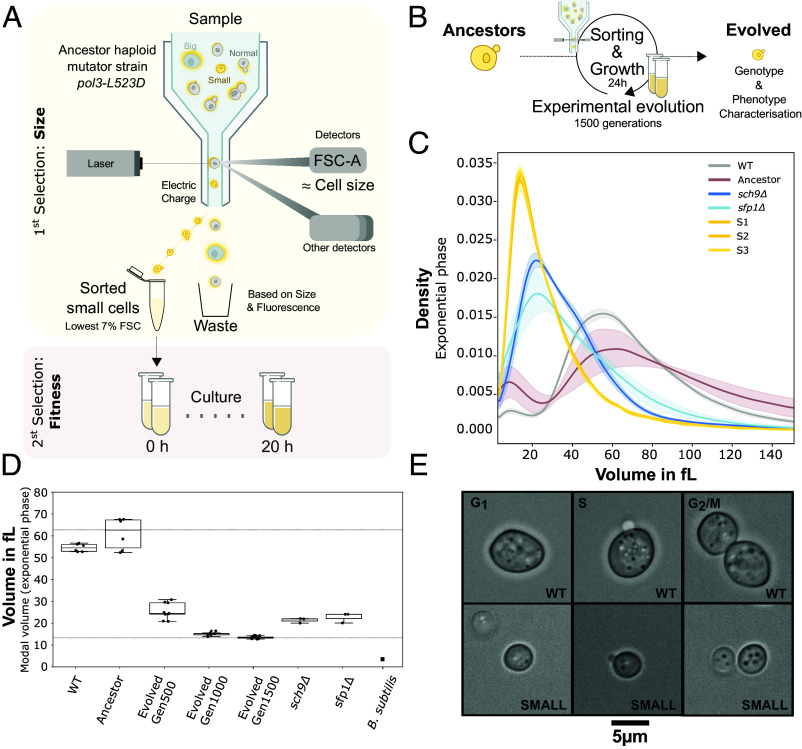
Experimental evolution of cellular miniaturization. Schematic representations of (*A*) size and fitness selection and (*B*) evolution regime. A haploid *pol3-L523D* mutator ancestor population was sorted into three small (S) subpopulations and subjected to daily size selection. Cells in the lowest 7% of the forward-scatter distribution were sorted (100,000 cells) and cultured in 10 mL of rich medium (YPD). Populations were serially grown and sorted for 116 cycles (~1,500 generations), with samples collected and stored every 24 h. (*C*) Cell volume distributions of exponential-phase cultures. Smooth density curves represent kernel density estimates fitted to histograms of cell volumes measured with a Coulter counter. For each strain, densities were computed per replicate and averaged to obtain a mean density curve. Shaded areas represent the standard deviation (SD) across replicates. In larger strains, the first peak corresponds to debris rather than intact cells, a known Coulter counterartifact that is captured as an artificial peak in the density estimate. (*D*) Modal cell volume from measurements of exponential-phase cultures across generations. Data from the three Small-populations are plotted together (Evolved), showing progressive evolution toward smaller size, reaching a fourfold volume reduction. Gray lines indicate ancestor and final evolved sizes. Box plots show medians, interquartile ranges (IQR), and whiskers (1.5× IQR). (*E*) Light micrographs of WT and evolved small-cell populations at different cell-cycle stages (G1, S, G2/M).

To investigate how cell size can evolve under sustained proliferative capacity, we developed an experimental system in *S. cerevisiae* that alternates two selective regimes targeting cell size and reproductive fitness, respectively. Cell populations were sorted by FACS, using the light scattered by cells (Forward Scatter Area, FSC-A), a widely used proxy for their size (46). Only the smallest 7% of cells were collected into separate tubes and subjected to the second selection step (Fig. 1*A*; see *SI Appendix* and *SI Appendix*, Fig. S1*A* for details). The second selection consisted of the unconstrained growth of the sorted population for at least 11 generations, during which cells competed for resources (Fig. 1*A*). After reaching marked opacity, typical of cells reaching stationary phase, the three parallel small (S1–S3) populations were subjected again to both selection regimes. Repeating this cycle daily resulted in approximately 1,500 generations of evolution (Fig. 1*B*). Under these conditions, selection favors mutations that reduce cell size without compromising proliferation. To accelerate evolution, the starting haploid population (ancestor) carried a mutator allele in the catalytic subunit of DNA polymerase delta [*pol3-L523D* (47), Dataset S1]. In addition, we engineered the ancestor strain to express a cytoplasmic fluorescent protein (ymCitrine) to prevent subcellular debris from being sorted.

Over 1,500 generations, the evolving populations (S1–S3) showed a gradual decrease in FSC values as measured by flow cytometry (*SI Appendix*, Fig. S1*B*). Microscopy and Coulter counter analyses confirmed that this reduction was accompanied by a marked decrease in cell volume (Fig. 1 *C*–*E*). Although genomic analyses later revealed partial cross-contamination among evolving lines, likely introduced during the sorting procedure, the final populations retained distinct clonal compositions and unique mutations, and were therefore analyzed independently. Literature values from Coulter counter measurements are typically reported from exponential-phase cultures, when cells divide rapidly in the presence of abundant nutrients. In contrast, in our experimental protocol, size selection occurred as cultures approached the stationary phase, when nutrients become limiting and cell division progressively slows.

To determine whether the evolved reduction in cell size was maintained across growth phases, we measured cell volumes in both exponentially growing and saturated cultures (Fig. 1 *C* and *D* and *SI Appendix*, Fig. S1 *C*–*E*). In exponential phase, WT and Ancestor cells exhibited unimodal size distributions with modes of 54.7 ± 1.6 fL (mean ± standard deviation (SD)) and 61.2 ± 7.29 fL (mean ± SD), respectively. By generation 1,500, the Small-populations displayed an approximately fourfold reduction in modal size over WT (12.8 to 13.8 fL; Fig. 1 *C* and *D*), accompanied by ~2.5-fold and ~2.6-fold decreases in mean and median volumes, respectively (mean ~36 vs. 91 fL in WT; median ~26 vs. 67 fL in WT), approaching volumes closer to those of the bacterium *Bacillus subtilis* than to their own ancestor. Comparatively, perturbations of the TOR signaling pathway (*sch9*Δ and *sfp1*Δ), which produce some of the smallest previously described yeast cells (35, 48), only reduce modal volumes by ~2.5-fold (22.8 ± 2.9 fL and 22.6 ± 1.9 fL mean ± SD, respectively, Fig. 1*C*). Upon entry into stationary phase, WT populations displayed a broader and frequently bimodal volume distribution, with peaks corresponding to small daughter cells (~22 fL) and larger mother cells (~80 fL) (*SI Appendix*, Fig. S1 *C* and *D*). In contrast, the size distributions of the evolved populations remained unimodal, with a single peak around ~11 fL. Because the WT stationary-phase distribution reflects a mixture of cell types, we compared median cell volumes, which revealed that the evolved lines remained approximately 2.5-fold smaller than WT cells, indicating a robust size reduction also at the end of the growth curve (*SI Appendix*, Fig. S1*E*). Unless otherwise noted, all cell size measurements reported below were obtained from exponentially growing cultures.

Together, these results indicate that, to the best of our knowledge, this selection experiment produced the smallest *S. cerevisiae* cells reported to date under standard laboratory growth conditions. Modal cell sizes as low as 16 fL have previously been reported for mutants affecting the G1/S transition when grown in poor carbon sources such as glycerol; however, these conditions markedly slow cell growth and division (20, 35, 49, 50) (*SI Appendix*, Fig. S1*F*). Comparable size reductions in *Schizosaccharomyces pombe* have been reported to cause mitotic catastrophe (51, 52), highlighting the severe constraints on extreme size reduction. Strikingly, the evolved cells reached volumes similar to those of the smallest species in the *Saccharomycotina* subphylum (*SI Appendix*, Fig. S1*G*), spanning nearly half of the natural size diversity observed across the subphylum, despite over 400 My of divergence (53).

### Gradual Cell Size Decrease Reshapes Cell Cycle Dynamics, Cellular Morphology, and Nutrient-dependent Size Control.

**Fig. 2.**
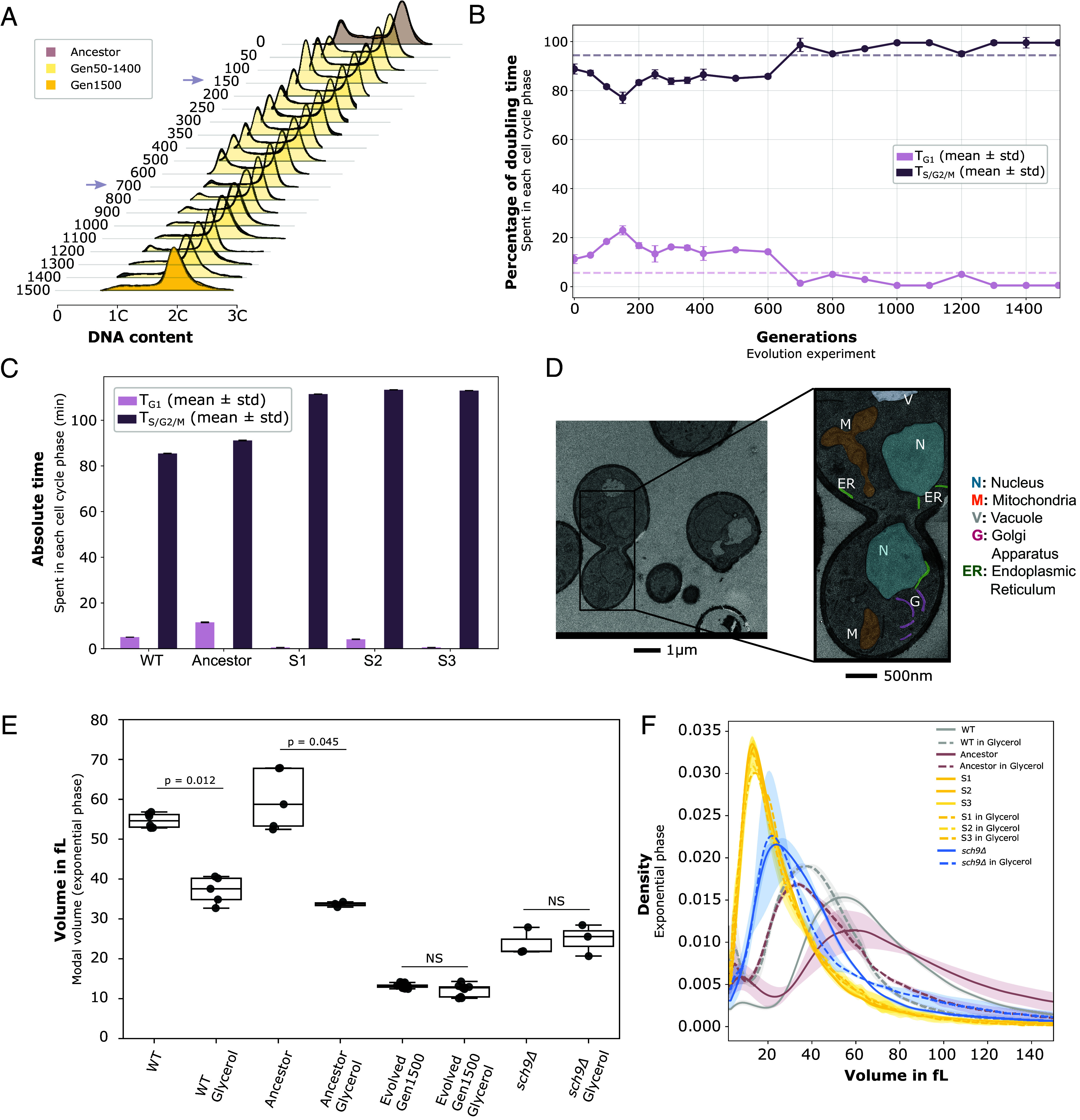
Physiology of cell miniaturization. (*A*) DNA-content profiles of ancestral and representative evolved (S1) populations during exponential growth across 1,500 generations, measured by flow cytometry. Lines show means; shaded contours, SD (n = 3). G2 peak height was normalized to 1. Arrows indicate time points of cell cycle profile changes (see main text). (*B*) Percentage of doubling time spent in G1 and S/G2/M phases by the S1 population across 1,500 generations. Bars show mean ± SD (n = 3). Dotted lines represent WT values. (*C*) Absolute time spent in each cell cycle phase, calculated as the fraction of the doubling time allocated to each phase (55). (*D*) Transmission electron micrograph of evolved *S. cerevisiae* cells during exponential growth in YPD. (*E*) Modal cell volume measurements of exponential-phase cultures grown in glucose or glycerol. Data from the three Small-populations are plotted together (Evolved). Box plots show medians, interquartile ranges, and whiskers (1.5× IQR). Pairwise comparisons between carbon sources (glucose vs. glycerol) were performed using the Mann–Whitney U test. (*F*) Cell volume distributions of exponential-phase cultures grown in glucose or glycerol as a carbon source. Smooth density curves represent kernel density estimates fitted to histograms of cell volumes. For each strain, densities were computed per replicate and averaged to obtain a mean density curve. Shaded areas represent the SD across replicates.

The dramatic changes in cell volume observed during our evolution experiment prompted us to examine potential alterations in cellular architecture. The cell cycle is a major determinant of cell morphology in budding yeast (54). To determine whether changes in cell size were associated with alterations in the cell cycle, we analyzed DNA content in exponentially growing populations, where signal intensity distinguishes cell cycle phases (1C for G1, 2C for G2/M, and 1C < DNA < 2C for S phase). Strikingly, by generation 1,500, evolved small cells displayed a near absence of G1 compared to WT (Fig. 2*A* and *SI Appendix*, Fig. S2*A*). Notably, cell cycle changes during evolution occurred in two steps: during the first ~150 generations, the proportion of cells in G1 increased, followed by a sharp shift around generation 700 that resulted in the near disappearance of G1 (Fig. 2*B* and *SI Appendix*, Fig. S2*B*). Calculating the absolute time spent in each phase confirmed this trend, showing that the reduction of G1 duration was primarily compensated by a corresponding extension of S/G2/M phases (Fig. 2 *B* and *C* and *SI Appendix*, Fig. S2*B*).

We next focused on intracellular organization, reasoning that reduced volume might occur at the expense of specific organelles. To obtain a general view of subcellular structures, we performed transmission electron microscopy (56) on exponentially growing WT, ancestral, and evolved Small-populations. Buds smaller than mother cells, consistent with asymmetric cell division (15), were commonly observed. Interestingly, major organelles displayed their typical morphology, though with altered proportions, suggesting a potential differential miniaturization of organelles (Fig. 2*D* and *SI Appendix*, Fig. S2*C*). Despite the presence of petite clones (cells lacking functional mitochondrial respiration) within the populations, most evolved isolates, including the smallest, retained growth in nonfermentable carbon sources, indicating preserved mitochondrial function. Petite clones did not differ in cell size from nonpetites (*SI Appendix*, Fig. S2*D*).

Given their preserved respiratory capacity, we tested whether evolved cells maintain the canonical relationship between nutrient quality and cell size. Typically, cells decrease their volume in lower-quality media (e.g., nonfermentable carbon sources) relative to high-quality media such as glucose. When cultured in the nonfermentable carbon source glycerol, WT and ancestor cells reduced their modal volumes from 55 and 60 fL to 37 and 34 fL respectively, corresponding to a ~1.5- to twofold decrease in modal cell volume. In contrast, evolved populations maintained almost identical modal cell sizes in glucose and glycerol (13.22 vs. 12.12 fL; Fig. 2 *E* and *F* and *SI Appendix*, Fig. S2*E*). This result suggests either that evolved cells have reached their minimal attainable cell volume, or that the molecular pathway linking nutrient quality to cell size has been altered to mimic poor-nutrient conditions even in high-quality media.

Together, these observations indicate that cellular miniaturization in evolved populations is accompanied by extensive remodeling of cell cycle progression, cellular organization, and nutrient-dependent size control.

### Evolved Cells Decouple Miniaturized Size From Pronounced Slow Growth.

**Fig. 3.**
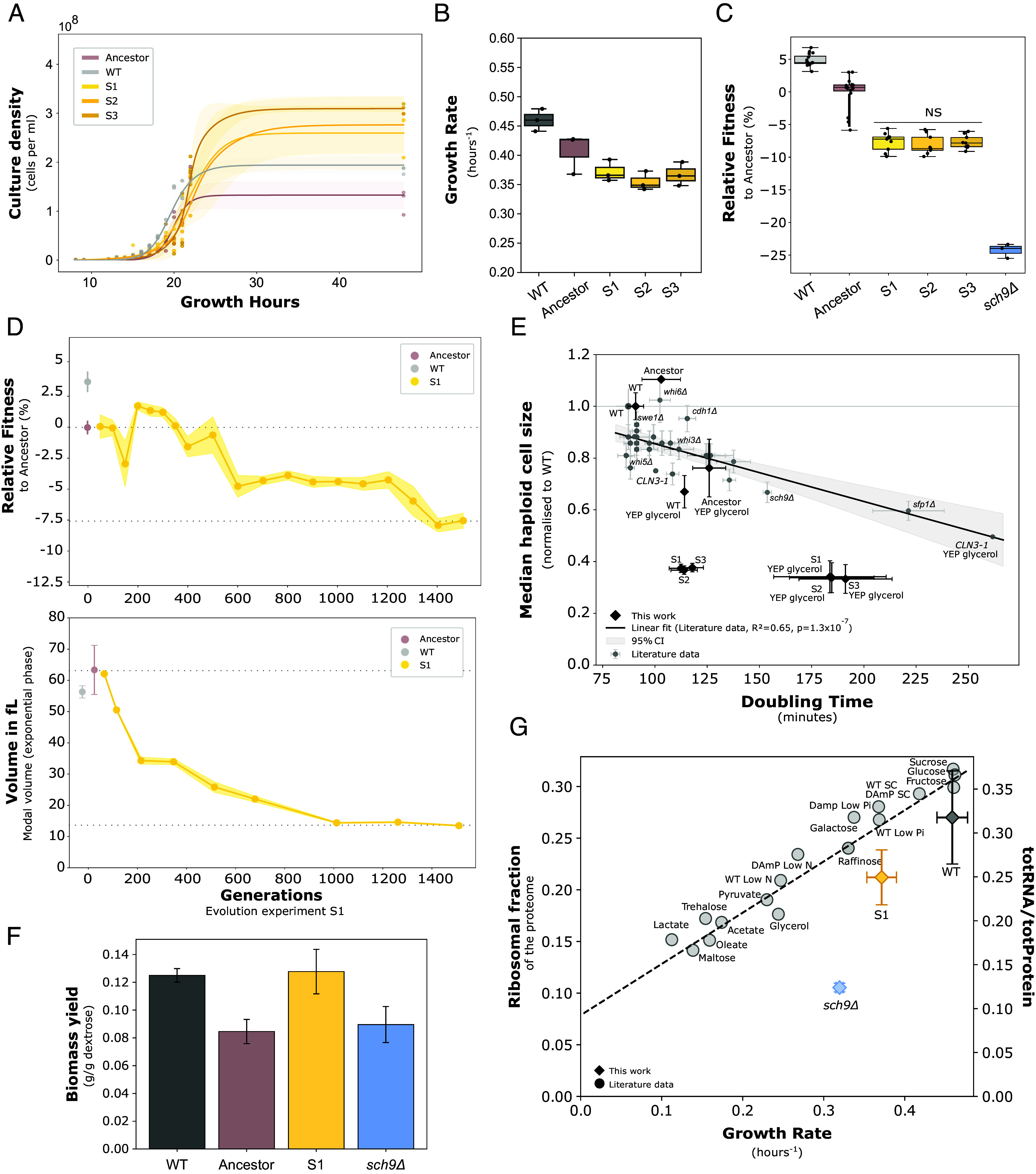
Fitness and biomass production. (*A*) Growth curves of selected strains measured as cell density (cells/mL) vs. time (h). (*B*) Maximum growth rates in YPD. Box plots show medians, interquartile ranges, and whiskers (1.5× IQR). (*C*) Fitness of WT and evolved populations (S1–S3) at generation 1,500, relative to the ancestor. Box plots as in *B*. Full pairwise statistics are provided in Dataset S7. (*D*) Fitness and cell size trajectories across 1,500 generations for the S1 population. Lines show mean values; shaded areas show SD. (*E*) Relationship between fold decrease relative to WT median cell size and doubling time in *S. cerevisiae*. Circles represent data from refs. 35, 49, and 50. Diamonds represent strains measured in this study. Error bars represent propagated measurement uncertainty. The solid black line shows a linear regression computed using only literature measurements, with the shaded area representing the 95% confidence interval (CI). Strains of interest are annotated. (*F*) Biomass derived from dry mass per cell, converted to yield relative to dextrose content in the medium. (*G*) Scaling of the ribosomal fraction of the proteome with population growth rate. Proteome-derived ribosomal fractions from ref. 58 (circles, left y-axis) are compared with RNA/protein ratios measured in this study (diamonds, right y-axis). These two quantities can be directly related, assuming that 85% of total cellular RNA corresponds to rRNA (59). A linear regression (dashed line) was fitted to the ribosomal fraction values reported in ref. 58.

Marked decreases in cell size, whether caused by mutations, poor nutrients, or a combination of both, are typically associated with pronounced slow growth. We therefore wondered how changes in cell size affected the evolved populations’ growth rates. Growth curves of WT, ancestral, and Small-populations revealed a marked increase in maximum population density in the evolved lines (Fig. 3*A*). Unexpectedly, their growth rates were only marginally reduced relative to ancestors, which themselves grew slower than WT, potentially due to the mutator phenotype (Fig. 3*B* and *SI Appendix*, Fig. S3 *A*–*C*). To precisely estimate this defect, we resorted to pairwise competition assays, which quantify relative fitness, a measure of reproductive success of organisms (57). All three evolved Small-populations had equivalent relative fitness (–7,79% ± 1.4, mean ± SD vs. Ancestor), whereas WT showed a ~5% advantage relative to the ancestor (Fig. 3*C*). In contrast, deletion of *SCH9*, which produces some of the smallest cells in rich media, caused a severe fitness defect (~24% vs. Ancestor & ~28% vs. WT) despite reducing modal cell volume by only 2.5-fold in exponential phase. Across all populations, fitness followed a nonuniform trajectory: i) declining during the first 200 generations, ii) recovering by generation ~250, iii) dropping sharply until ~500, and iv) stabilizing with minor decreases until generation 1,500 (Fig. 3*D* and *SI Appendix*, Fig. S3 *D*–*G*). Fitness was more strongly associated with cell-cycle dynamics than with cell volume (Pearson r = 0.67, *P* < 0.001 for G1 fraction; r = 0.30, *P* = 0.11 for cell volume), indicating that changes in fitness primarily reflect alterations in cell-cycle progression (Figs. 2*B* and 3*D*). Importantly, these results highlight how evolved lines fall outside of an inverse linear relationship between cell size and doubling time observable across a wide range of mutants (Fig. 3*E*).

The most pronounced decreases in cell size have previously been associated with nutrient limitation or mutations in nutrient-sensing pathways. These conditions were proposed to modulate cell size through reduced ribosome biogenesis (48). However, numerous microbial physiology studies have linked reduced ribosome biogenesis with decreased growth rates (60, 61). This raises the question: how do evolved cells maintain a miniaturized cell size while sustaining growth rate and fitness? To address this, we examined the biosynthetic capacity of the evolved lines. At saturation, the dry mass produced per gram of dextrose by evolved lines was comparable to that of WT, whereas *sch9Δ* cells produced approximately a third less (Fig. 3*F*). We next estimated the ribosomal fraction of the proteome from the RNA/protein ratio, a commonly used proxy for ribosome biogenesis in microbial physiology, also applicable to yeast (61). As expected, *sch9Δ* cells showed a marked reduction in ribosomal mass fraction, whereas the evolved lines maintained ribosome biogenesis at levels closer to WT (Fig. 3*G*).

Altogether, these results suggest that evolved lines maintain efficient growth despite a markedly reduced cell volume, by preserving ribosome biogenesis and biomass production.

### New Evolved Size Homeostasis Is Stable and Robust.

**Fig. 4.**
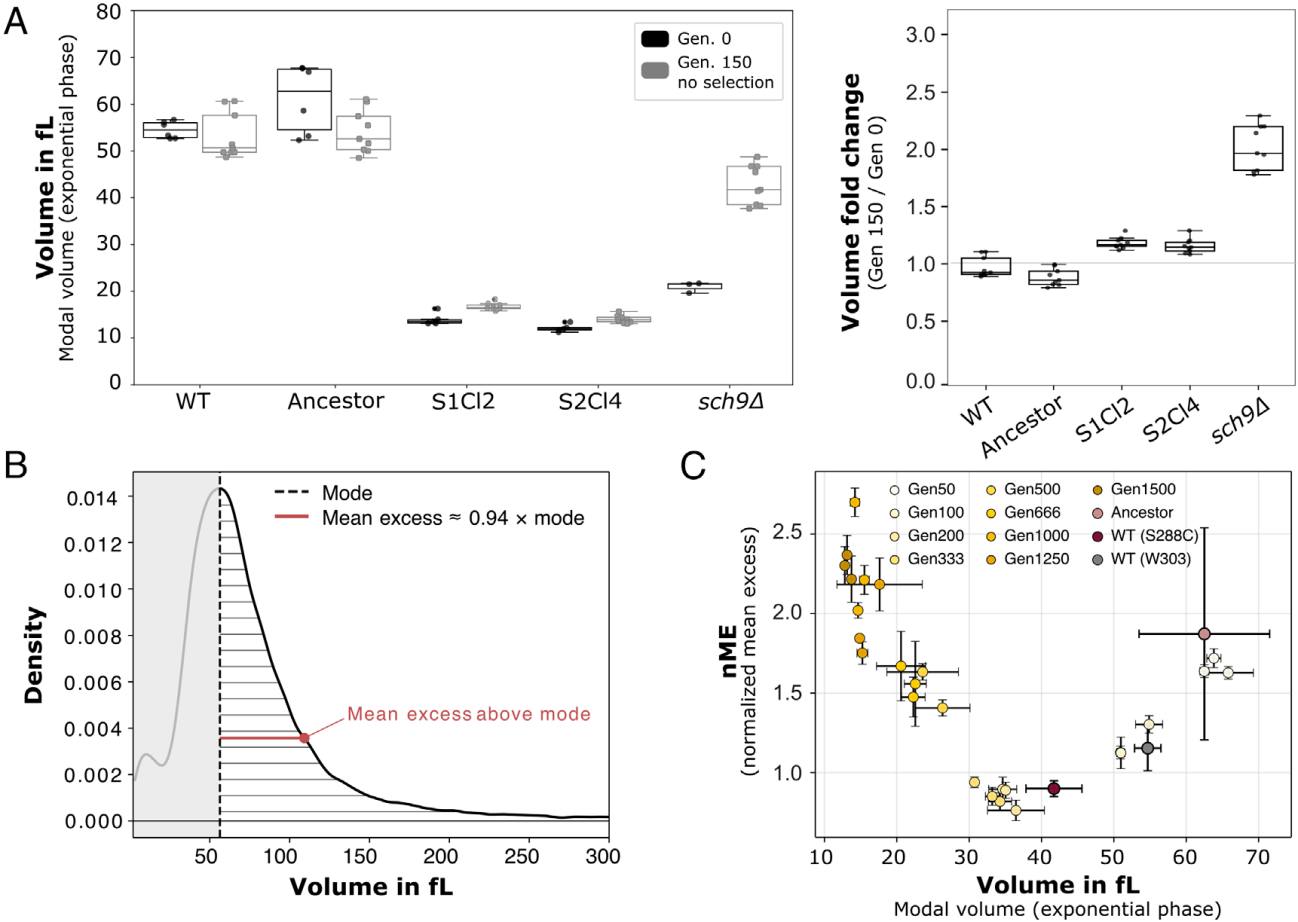
Evolution of cell size homeostasis. (*A*) Stability of cell size during unconstrained experimental evolution. *Left*: cell size measurements of WT, Ancestor, *sch9Δ*, and evolved clones before and after 150 generations of evolution without size selection. *Right*: fold change in cell size during evolution in the absence of size selection. Box plots show medians, interquartile ranges, and whiskers (1.5× IQR). (*B*) Our metric of cell size distribution width calculates the mean excess above the mode, which is a generalization of the half-width at half maximum that accounts for the distribution width across all density values, rather than just the half maximum (*SI Appendix*). We focus on the right tail of the distribution to avoid spurious effects due to the presence of cell debris at small cell volumes. Shown here is the distribution of WT (W303). (*C*) Width of cell size distributions, quantified as the mean excess above the mode (*SI Appendix*), normalized by modal volume. The Ancestor (pink, ~62.5 fL modal volume, normalized mean excess (nME) ~2.8) had a broad right tail relative to WT, leading to a large nME.

How stable is the evolved miniaturized size? Measurements performed right after the last sorting round may reflect a transient physiological state rather than an evolutionarily stable phenotype. To address this possibility, clones were isolated from two independent populations (S1Cl2 and S2Cl4) and passaged for 150 generations in the absence of any size selection. Interestingly, Small-clones stably maintained their size across the experiment, resulting in a final fold change similar to that observed in control WT and ancestor lines (Fig. 4*A* and *SI Appendix*, Fig. S4 *A*–*D*). In contrast, *sch9*Δ lines consistently reverted to sizes closer to WT, despite starting with a larger volume than Small-clones (Fig. 4*A* and *SI Appendix*, Fig. S4 *A*–*D*).

A key question is whether miniaturized cells actively regulate their size, or whether they merely preserve the volumes attained at the end of the experiment. To address this, we transiently arrested cells at the G1/S transition using the pheromone α-factor, which allows growth while blocking division (62) (*SI Appendix*, Fig. S4 *E* and *F*). While the arrest was not fully efficient in Small-clones, it nevertheless led to a ~1.8-fold increase in cell volume in both WT and evolved cells. The increased volume, however, was progressively reversed upon release into fresh medium, indicating that cells actively return to their characteristic small size (*SI Appendix*, Fig. S4 *E* and *G*).

The ability of miniaturized cells to maintain their size across many generations and under transient perturbations reflects efficient size homeostasis, which is expected to manifest as a narrow distribution of cell sizes around the population mean. To test this prediction, we compared the width of cell size distributions across WT, ancestral strains, and evolved populations at successive generations Fig. 4 *B-C*. To avoid spurious contributions from cell debris and noncellular particles at low volumes, which bias conventional statistics such as the mean and SD, we quantified distribution width as the mean excess above the modal volume $E[v-v_{\mathrm{mode}}|v\geq v_{\mathrm{mode}}]$, normalized by the modal volume (*SI Appendix*). This dimensionless metric captures the average relative width of the right tail of the size distribution independently of debris at low volumes (Fig. 4*B*). Over the course of evolution, populations initially reduced both their modal volume and their normalized mean excess (nME), converging toward values below those of the ancestor and comparable to WT (Fig. 4*C*). Once modal volumes fell below ~30 fL, however, nME increased again, returning to values comparable to the ancestor despite a dramatically reduced characteristic cell size. Overall, miniaturized Small-clones thus maintain a degree of size homeostasis similar to the ancestor, demonstrating that the reduction in cell size was not accompanied by a proportional loss of size control.

Together, these results demonstrate that miniaturized cells have evolved a new cell size homeostasis that is evolutionarily stable, robust to transient perturbations, and capable of maintaining population-level size control as tightly as their own ancestor.

### Genetic Landscape of Cellular Miniaturization.

We sought to determine the genetic basis of the evolved phenotype. Evolved cells retained their characteristic size when cocultured with WT strains, indicating that miniaturization was not driven by secreted or diffusible factors (*SI Appendix*, Fig. S5*A*). The phenotype’s persistence over 150 generations in the absence of selection (Fig. 4*A*) strongly suggested a genetic basis. Supporting this idea, crosses between evolved clones and WT produced diploids of intermediate size between haploid and diploid WT, consistent with the presence of both recessive and dominant mutations. Furthermore, crosses between evolved clones and mating-type–switched clones yielded diploids that retained a large reduction in cell volume relative to WT diploids (*SI Appendix*, Fig. S5*B*), suggesting that the reduced cell size is independent of ploidy.

**Fig. 5.**
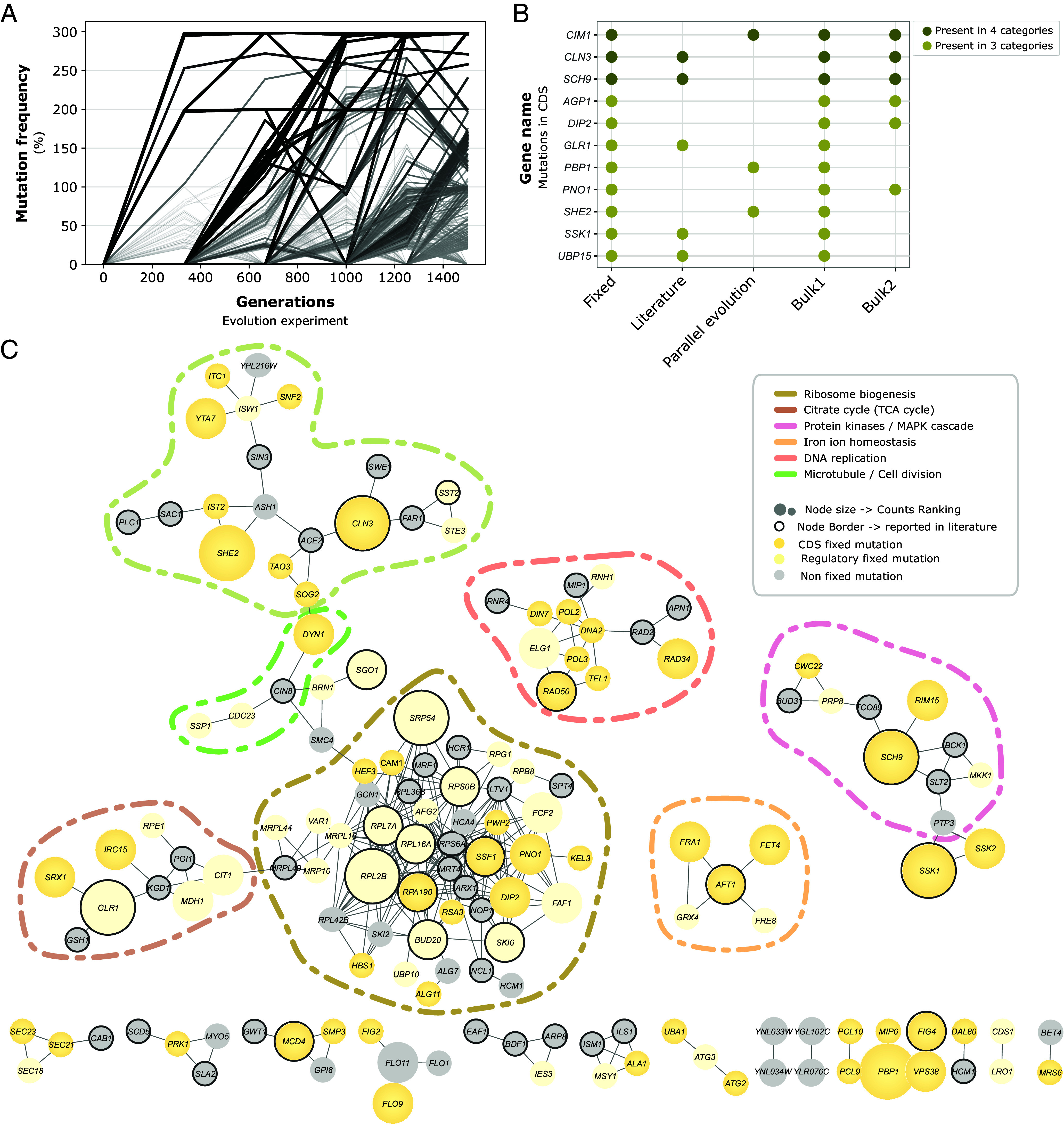
Genomic landscape of the evolved phenotype. (*A*) Allele-frequency trajectories of mutations detected in the combined population (S1–S3). A 300% frequency indicates fixation in all three populations. Line thickness reflects the final frequency of the mutation at generation 1,500. (*B*) List of putative adaptive gene mutations ranked by the number of satisfied criteria outlined in the main text and affected by nonsynonymous mutations located within coding sequences (CDS). Only genes appearing in at least three categories are included (full list in Dataset S6). (*C*) Interaction network of mutations detected in S1–S3 evolved populations curated in Cytoscape (63). Gray lines indicate known genetic and physical interactions [STRING database (64)]. Node diameter reflects the number of satisfied criteria we used to detect putative adaptive mutations. Only genes appearing in at least two categories are included. Nodes are color-coded: dark yellow for nonsynonymous mutations within CDS fixed at generation 1,500, light yellow for mutations in the regulatory regions fixed at generation 1,500, and gray for nonfixed mutations. Bold outlines mark genes linked to cell size changes in the literature (either decrease or increase).

To identify causative mutations, we performed whole-genome, whole-population sequencing across multiple evolutionary timepoints. As expected from the mutator allele present in the ancestral population, we detected 869 new unique mutations that arose during evolution in coding and regulatory regions across the three populations (Fig. 5*A* and Dataset S2). By the end of the experiment, an average of 267 single-nucleotide polymorphisms (SNPs) and small insertions/deletions (INDELs) persisted per population (285 in S1, 301 in S2, and 216 in S3, *SI Appendix*, Fig. S5*C*). No aneuploidies, segmental amplifications, or large-scale changes in DNA copy number were observed, thus confirming the presence of a full haploid genome (*SI Appendix*, Fig. S5*D*). The evolved populations exhibited distinct clonal compositions (*SI Appendix*, Fig. S5*E*); however, several alleles were shared across populations. These mutations were absent from the ancestral strain and, in most cases, involved identical nucleotide substitutions. The probability that a large number of identical mutations arose independently across populations is extremely low, therefore pointing toward a likely cross-contamination during the FACS selection steps. Accordingly, the evolved lines were treated as a single evolving population for subsequent genomic analyses rather than as independent replicates (Fig. 5*A*).

To identify putative adaptive mutations associated with the evolved cell size, we employed four nonexhaustive, but complementary approaches: i) tracking lineages that rose to fixation in any of the three subpopulations, identifying cohorts of mutations under positive selection (Dataset S2); ii) searching for genes previously implicated in cell size regulation (Dataset S3); iii) using statistical inference to identify genes repeatedly targeted by independent nonsynonymous mutations across evolved populations more frequently than expected by chance (parallel evolution; Dataset S4); and iv) performing two consecutive rounds of backcrossing and selection for small cell size to pinpoint mutations segregating with the phenotype (bulk segregant analysis; Dataset S5; see *SI Appendix* for details). A summary of the genes identified by these methods, as well as their reported interactions, is portrayed in Fig. 5 *B* and *C* (Dataset S6). Despite several genetic and physical interactions among the identified genes, no gene ontology term was found to be statistically enriched.

Our results indicate that the miniaturized phenotype is heritable and genetically encoded, but the underlying adaptive landscape is complex. The large number of candidate mutations likely includes false positives arising from hitchhiking or linkage, as well as variants under indirect selection. Some of these may reflect selection on traits influencing the flow-cytometry size proxy (FSC-A) rather than cell size itself, or compensatory adaptations to physiological changes associated with miniaturization (65).

### Combined Mutations in the G1 Cyclin and Greatwall Kinase Signaling Cascades Cause Large Deviations in Cell Size.

**Fig. 6.**
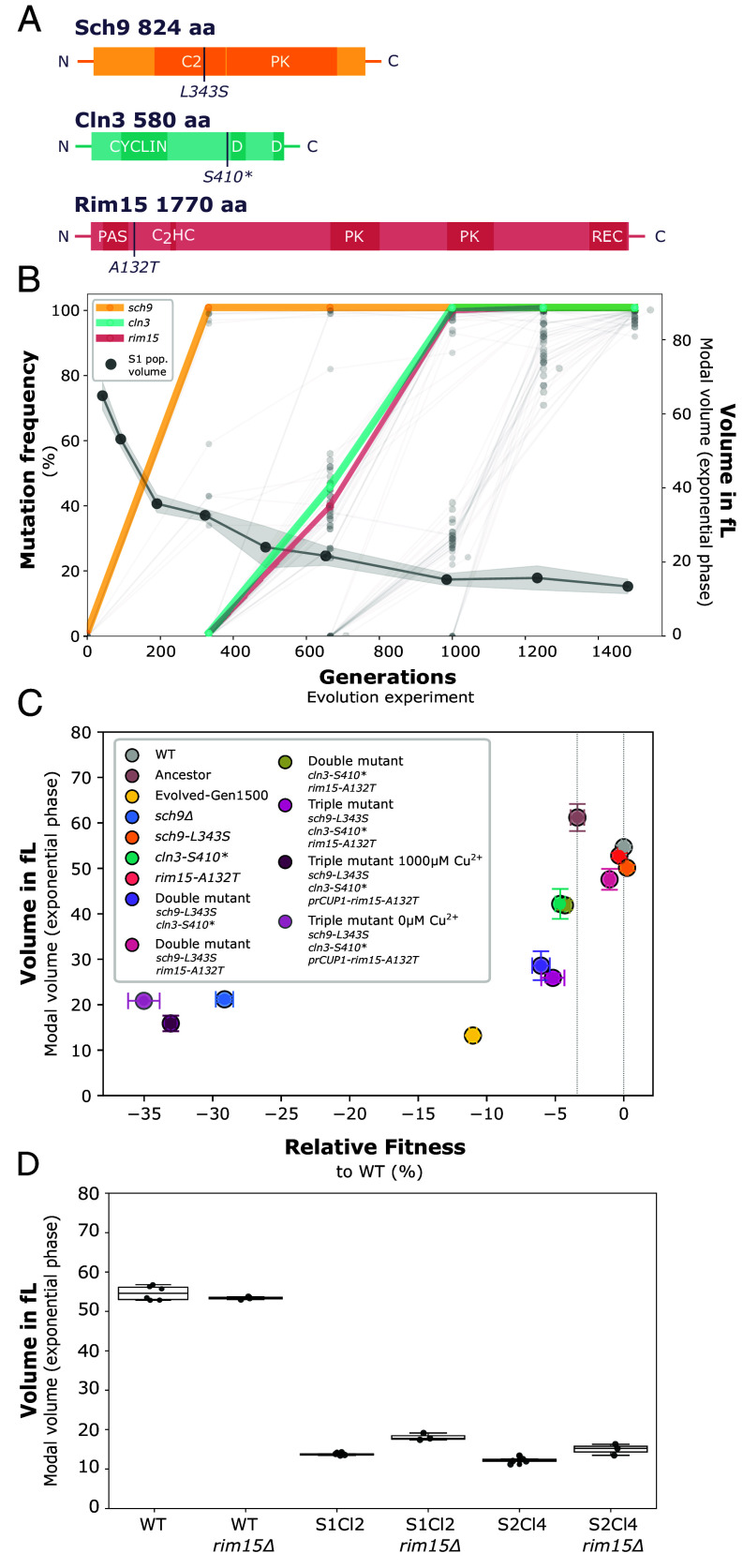
Genetic basis of the cell size phenotype. (*A*) Schematic of putative adaptive mutations. Protein domains are indicated, and mutations identified during evolution are marked: PK, kinase catalytic domain; D, consensus disorder prediction; PAS, N-terminal PAS domain; C2HC, CCHC-type zinc-finger domains; REC, C-terminal receiver domain. (*B*) Allele-frequency trajectories of putative adaptive mutations. Only variants reaching ≥80% frequency by generation 1,500 are shown; causative mutations are highlighted. The right y-axis shows the S1 population volume (circles) over time. (*C*) Relationship between relative fitness and modal cell volume. Fitness (x-axis) and cell volume (y-axis) are shown; error bars indicate SD. (*D*) Modal cell volume following deletion of *RIM15* in WT and final evolved clones, from exponential-phase cultures. Box plots show medians, interquartile ranges, and whiskers (1.5× IQR).

We set out to identify the mutations primarily responsible for the change in cell size among those identified as putatively adaptive. *CIM1*, *CLN3*, and *SCH9* ranked highest in our analysis and were therefore selected for further investigation. *CIM1* encodes for a mitochondrial HMG-box protein that limits mitochondrial DNA copy numbers (66), and independently accumulated up to six mutations in the protein over the course of the experiment, including three frameshifts resulting in premature stop codons (*SI Appendix*, Fig. S6*A*). Cln3, a G1 cyclin that partners with Cdk1 to drive the G1/S transition by activating phase-specific gene expression, has been shown to reduce cell size when truncated at its C terminus (e.g., *CLN3-1*), due to increased protein stability (49, 67, 68). Our evolved strains carried a similar truncation, cln3-S410*, just downstream of the *cln3-1* allele (Fig. 6 *A* and *B*). Sch9 is a key effector of the TORC1 pathway, which integrates nutrient sensing with anabolic and catabolic processes (69). We identified a mutation (*sch9-L343S*) within the conserved C2 domain (residues 184 to 402), a calcium-dependent membrane-targeting module that may also inhibit the kinase domain [residues 403 to 738; Fig. 6 *A* and *B*, (70)].

To test the causality of these mutations, we reconstructed the *cim1*, *cln3*, and *sch9* alleles in a WT background. Deletion of *CIM1*, used to mimic the observed frameshift mutations, did not alter cell size (*SI Appendix*, Fig. S6*A*), suggesting a potential adaptive role in other cellular features or fitness. In contrast, mutations in *CLN3* and *SCH9* individually caused a modest but significant decrease in cell volume (Fig. 6*C*). Combining the two mutations produced synergistic effects, with the double mutant exhibiting a threefold reduction in modal volume (28.5 fL; Fig. 6*C*).

Truncations of the C-terminal region of Cln3 confer a gain-of-function phenotype by increasing protein stability and cyclin abundance (68). Loss-of-function mutations in *SCH9* have likewise been associated with reduced cell size, typically attributed to its role in ribosome biogenesis (48). However, the elevated ribosome biogenesis observed in evolved lines (Fig. 3*G*), together with their sustained fitness (Fig. 3 *B* and *C*), indicates that *sch9-L343S* is a separation-of-function allele that affects cell size through a mechanism distinct from ribosome biogenesis.

Recent work suggests that Sch9 can also regulate cell size via the Greatwall kinase Rim15, albeit under conditions of low CDK activity (71). This pathway is highly conserved among eukaryotes and controls cell cycle progression in metazoans through endosulfine-mediated inhibition of the phosphatase PP2A (72, 73). While it links nutrient sensing to cell size in *S. pombe* (74), in *S. cerevisiae* it has primarily been implicated in the regulation of quiescence (75). We identified mutations across multiple components of this pathway, including a fixed mutation in *RIM15*, as well as lower-frequency mutations in the promoter of the endosulfine *IGO1* and in *CDC55*, which encodes a regulatory subunit of PP2A (76). Reconstruction of the evolved *RIM15* allele (*rim15-A132T*) resulted in a modest, nonsignificant reduction in cell volume (Fig. 6*C*). However, the distribution of mutations across the pathway (*sch9-L343S*, *rim15-A132T*, *igo1-T-75G*, and *cdc55-L159P*; *SI Appendix*, Fig. S6*B*) suggests that the Greatwall pathway activity was modulated during evolution. To test this hypothesis, we engineered a copper-inducible tunable promoter (*prCUP1*) to control expression of *RIM15* and *rim15-A132T*. Copper titration revealed a clear dosage-dependent effect: basal expression reduced cell size, and overexpression induced further shrinkage in WT backgrounds (*SI Appendix*, Fig. S6*C*). Notably, overexpression of *rim15-A132T* in the sch9-L343S cln3-S410* background further reduced modal cell volume, matching that of the evolved lines (16 ± 0.1 fL, mean ± SD; Fig. 6*C* and *SI Appendix*, Fig. S6 *C*–*F*). Consistent with increased Greatwall pathway activity, deletion of *RIM15* increased exponential-phase cell volume in evolved clones but had little effect in WT (Fig. 6*D* and *SI Appendix*, Fig. S7*A*). In stationary phase, however, *RIM15* deletion increased cell size in both backgrounds, with a stronger effect in evolved cells (*SI Appendix*, Fig. S7 *B*–*D*). Furthermore, combined deletions of *RIM15* and *CLN3* showed synergistic effects on increasing WT cell size in stationary phase (*SI Appendix*, Fig. S7 *E*–*J*).

Importantly, despite their reduced size, all reconstructed strains carrying evolved alleles retained near-WT fitness (Fig. 6*C*). Although enforced expression of *rim15-A132T* phenocopied the size of evolved clones in the sch9-L343S cln3-S410* background, it imposed a fitness penalty. This penalty is unlikely to arise from reduced cell size itself, as fitness improved while size decreased further at higher copper concentrations (Fig. 6*C*), and instead points to deleterious consequences of constitutive Rim15 activation across the cell cycle. These observations suggest that, rather than simply increasing pathway output, evolution selected for combinations of mutations that tune Greatwall signaling in a context-dependent manner to minimize fitness trade-offs.

These results demonstrate that cell size in the evolved lines is dependent on Rim15 dosage and support a model in which, together with a hyperactive G1 cyclin Cln3, increased Greatwall pathway activity contributes to a reduced cell volume, although the precise molecular mechanisms remain to be defined.

## Discussion

How can evolution produce large shifts in cell size without incurring the detrimental effects associated with even small deviations from the norm? In development, abrupt changes often arise through ploidy increase or reduction (77). Linear scaling relationships between DNA content and cell volume are also observed across diverse taxa (78). Ploidy increases are important in plant evolution and likely account for part of the variation in cell size among related species (79). Yet, genome duplications alone cannot explain the wide diversity of cell sizes, as they also create barriers to sexual reproduction and would leave more pervasive genomic signatures if they were the main driver. Cells must therefore be able to modify their size independently of DNA content, at least initially, especially under gradual selection for divergent sizes.

Decades of studies on eukaryotic cell cycle regulation have identified the main principles allowing cells to regulate their size, as well as many molecular players involved (reviewed in refs. 11 and (80)). Mutations in many such players have been demonstrated to produce altered sizes (35, 36, 81). It is therefore intuitive to imagine that evolution could modulate cell size by fine-tuning these molecular players. Yet most such mutations reduce fitness, and it remains unclear which, if any, combinations of the dozens of known regulators could provide viable evolutionary paths to new cell sizes.

**Fig. 7.**
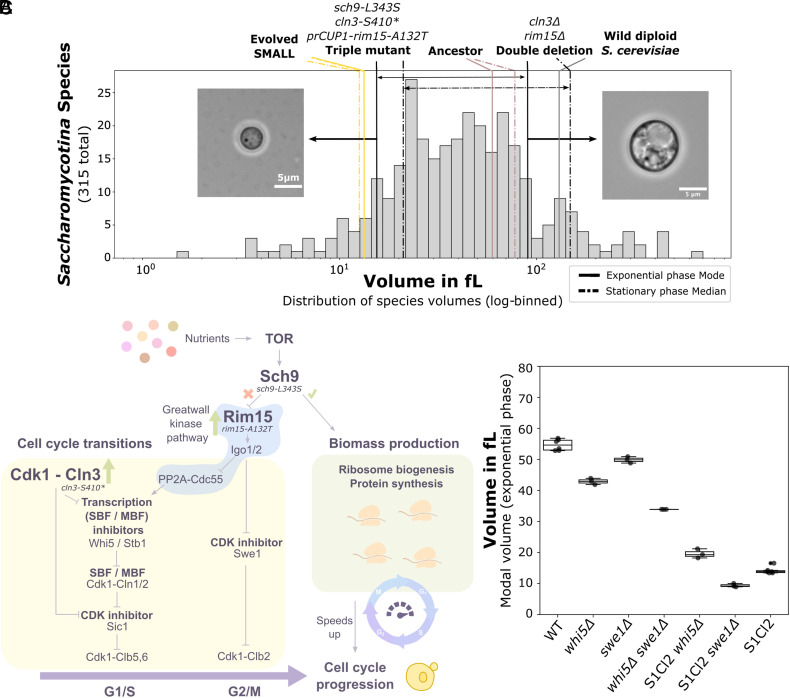
Speculative model of the effect of the identified mutations on cell cycle regulation. (*A*) Distribution of cell volumes across the *Saccharomycotina* subphylum (derived from ref. 53). Highlighted are the triple mutant (sch9-L343S cln3-S410* *prCUP1-rim15-A132T*) under a copper-inducible promoter and the *CLN3* and *RIM15* double deletions, showing shifts in size relative to natural variation. (*B*) Proposed mechanism: In WT cells growing under nutrient-rich conditions, TOR is active, promoting ribosome biogenesis and biomass accumulation (*Right*, green rectangle) while delaying cell cycle transitions (*Left*, orange rectangle). This results in fast-growing, large cells. Under nutrient limitation or in TOR-related mutants, Sch9 repression of Rim15 is relieved, leading to earlier cell cycle transitions. However, the accompanying reduction in biomass production limits growth and fitness. We propose that the combined effect of the identified mutations accelerates cell cycle transitions through a truncation in Cln3, loss of Sch9 inhibition, and gain-of-function mutations in the Greatwall kinase cascade, while maintaining ribosome biogenesis and biomass production. As a result, cells grow rapidly but, by spending less time in interphase, remain smaller than their WT counterparts. (*C*) Modal cell volume of *WHI5* and *SWE1* deletions in WT and final evolved clones. Box plots show medians, interquartile ranges, and whiskers (1.5× IQR).

We addressed this question experimentally by imposing selection on *S. cerevisiae* cell size and fitness. Over 1,500 generations, cells shrank by fourfold in modal volume compared to WT, without changing ploidy and with minimal fitness costs. We show that miniaturized cells maintain an efficient and evolutionarily stable cell size homeostasis, despite a significantly altered cell cycle progression characterized by the near absence of a G1 phase. Whole-genome sequencing revealed a complex genomic landscape, due to the mutator allele employed, and likely linked to the many direct and indirect changes cells experienced during miniaturization. Nevertheless, re-engineering putative adaptive alleles showed that simultaneous perturbations of the G1 cyclin Cln3 and the TOR effector Sch9 recapitulate much of the evolved phenotype with minimal fitness cost (Fig. 6*C*). Although consistent with a gain-of-function effect from C-terminal truncation of Cln3, our data do not support a primary role for Sch9 in size control via ribosome biogenesis (Fig. 3*G*). We argue instead for its involvement as an inhibitor of Rim15. Together, manipulations in the G1 cyclin and Greatwall kinase signaling cascades produced a sixfold size range within an otherwise constant genome, capturing up to 70% (221/315) of the natural variation in size seen across *Saccharomycotina* over 400 My of evolution (Figs. 6*C* and 7*A* and *SI Appendix*, Fig. S7 *E*–*J*).

Three questions arise. First, how can our evolved cells reach smaller sizes than those achieved previously through gene deletions? We propose that cell size evolution can proceed through combinations of modest-effect mutations affecting distinct cellular processes that converge on cell size control, thereby enabling substantial size reduction without the major fitness costs expected from strong perturbations of single regulators. We speculate the following model to account for the phenotype observed (Fig. 7*B*): truncations in *CLN3* stabilize the protein, increasing CDK1–Cln3 activity and phosphorylation of the transcriptional inhibitor Whi5 (82). In parallel, loss of Sch9 inhibition and gain-of-function mutations in the Greatwall kinase cascade inhibit the phosphatase PP2A–Cdc55, further driving Whi5 phosphorylation and nuclear exclusion (70). Why would this lead to a volume smaller than *whi5*Δ, where the inhibitor is absent? First, CDK1-Cln3 levels could simultaneously hyperphosphorylate the transcriptional regulator Stb1 (20, 83, 84) and accelerate the degradation of the CDK1 inhibitor Sic1 (85–87), thereby eliminating the G1 phase. Furthermore, *RIM15* activity in mitosis has been reported to destabilize the CDK1 inhibitor and size/morphogenesis checkpoint Swe1 (88), offering an additional route to reduced volume. To test this model, we deleted *WHI5* and *SWE1*, inhibitors of the G1/S and G2/M transitions respectively, in evolved clones (Fig. 7*C* and *SI Appendix*, Fig. S8*A*). *WHI5* deletion increased cell size in evolved clones (19.3fL), whereas *SWE1* deletion caused a stronger size reduction than in WT (33% vs. 9% decrease), producing cells with a modal volume of ~9 fL. These results indicate that *whi5*Δ no longer contributes to size decrease in evolved lines, while Swe1 plays a more prominent role, suggestive of cell size regulation occurring in G2/M rather than G1/S.

The second question regards what allows these miniaturized cells to retain high fitness. Eukaryotic cells physiologically shrink upon nutrient scarcity (13). Under these conditions, cells may find it advantageous to increase population-wide survival by dividing at smaller volumes while making a parsimonious use of the resources left by reducing ribosome biogenesis. The deletion of *SCH9* hard-wires this response, resulting in small cells with severe fitness defects due to strongly decreased protein production. Here, we show that our evolved lines do not shrink further in poor-quality nutrients. Because we achieved even smaller cells through the deletion of Swe1, we conclude that the evolved lines have genetically altered the mechanism linking nutrient quality to cell size, rather than reaching a minimal size limit. Although such a mechanism has been proposed to operate through the modulation of ribosome biogenesis, our results show that ribosome biogenesis remains efficient in our evolved lines. This indicates that the effect of nutrients on cell size has been decoupled from their effect on biomass production. This decoupling explains the high fitness of these evolved miniaturized cells and challenges current models of nutrient control on cell size.

Finally, have the evolved cells reached the lower size limit for *S. cerevisiae*? Gain-of-function mutants of the G1 cyclin Cln3, when grown in glycerol, approach the size of our evolved lines [16 fL, (49)]. This observation supports our model in which the evolved lines combine size effects from the G1 cyclin pathway with those mediated by nutrient signaling. However, we argue that this combined effect does not represent the lower physical limit, as the additional deletion of *SWE1* further reduces modal cell volume to ~9 fL. The long-term continuation of this evolutionary experiment will help determine whether, and how, a physical lower size limit can be reached experimentally.

In summary, our work shows that large deviations in cell volume can be achieved while maintaining cellular fitness and size homeostasis. We show that this phenomenon is accompanied by changes in cell cycle regulation and nutrient-dependent control of cell size. To achieve this phenotype, we propose a mechanism based on the simultaneous gain of function of the G1 cyclin and Greatwall kinase signaling pathways. In this model, increased Greatwall pathway activity arises, at least in part, from the relief of Sch9-mediated inhibition, thereby linking nutrient signaling to cell cycle control. We speculate that this model identifies a simple yet powerful route for evolution to achieve large deviations in volume without altering ploidy. Future studies should test the generality of the principles we identified and determine how broadly it underpins size diversity across the tree of life. The range of cell sizes generated within a single species, while maintaining high fitness and stable ploidy, also offers a framework to study subcellular scaling and the limits of cell size.

## Materials and Methods

All strains used in this study were W303 derivatives. Yeast cultures were propagated using standard techniques (89). Unless otherwise stated, experiments were performed in standard rich medium (YPD). Experimental evolution followed the procedure described in ref. 90, with the addition of the size-selection step by sorting, as detailed in the *SI Appendix*. Growth-curve measurements and fitness assays were performed as described in ref. 91. Samples for cell-cycle analyses were processed as in ref. 91 and analyzed as in ref. 55. Whole-genome sequencing and analysis followed procedures described in ref. 92. Extended methods are provided in *SI Appendix*.

## Supplementary Material

Appendix 01 (PDF)

Dataset S01 (XLSX)

Dataset S02 (XLSX)

Dataset S03 (XLSX)

Dataset S04 (XLSX)

Dataset S05 (XLSX)

Dataset S06 (XLSX)

Dataset S07 (XLSX)

## Data Availability

Genomic data, Scripts used for data analysis, and Raw Coulter Counter measurements data have been deposited in European Nucleotide Archive (ENA) (PRJEB101156) (93), GitHub (https://github.com/FumaLab/) (94), and Zenodo (https://doi.org/10.5281/zenodo.19254220) (95), respectively. All other data are included in the manuscript and/or supporting information.
