# Supplementary material for: Experimental evolution of cellular miniaturization reveals a putative mechanism for cell size evolution
Source: Proc Natl Acad Sci U S A. 2026 Jun 12;123(24):e2531280123. doi: 10.1073/pnas.2531280123 (PMC13273275; doi:10.1073/pnas.2531280123)
Supplement: Supplementary file 1 — Appendix 01 (PDF) [file pnas.2531280123.sapp.pdf]

PNAS

Supporting Information for

Experimental evolution of cellular miniaturization reveals a putative mechanism for cell size evolution

Ana Garoña[1], Morgane V. Lemos[1], Andrea Giometto[2], Marco Fumasoni[1]

[1] Gulbenkian Institute for Molecular Medicine (GIMM), Oeiras 2780-156, Portugal

[2] School of Civil and Environmental Engineering, Cornell University, Ithaca, NY 14853

Email: marco.fumasoni@gimm.pt

This PDF file includes:

Extended methods
Figures S1 to S8
Legends for figures S1 to S8

Extended methods

Strains and culture conditions

All strains used in this study were derived from *Saccharomyces cerevisiae* W303 (*leu2-3,112 trp1-1 can1-100 ura3-1 ade2-1 his3-11,15, RAD5*$^{+}$) and are listed in Dataset S1. The haploid strain yMF1001 (*ADE2; leu2-3,112; ura3-1; trp1-1; RAD5*$^{+}$*; yel068c::CAN1/URA3; prACT-Citrin-LEU2; pol3-L523D*) was used as the ancestral strain in the evolution experiments. This strain carries the *pol3-L523D* mutator allele, which lacks the proofreading activity of the main replicative polymerase and increases the mutation rate by approximately 100-fold (1). Yeast propagation was carried out using standard techniques (2). Unless otherwise specified, all experiments were performed in standard rich medium (YPD), consisting of 1% yeast extract, 2% peptone, and 2% D-glucose, supplemented with 0.01% adenine and tryptophan. For the evolution experiments, the medium was further supplemented with penicillin and streptomycin. Auxotrophic selection and complementation assays were performed in Synthetic Complete (SC) medium or in the appropriate dropout variants. SC medium consisted of 10% (v/v) 10× Yeast Nitrogen Base (YNB) without amino acids, supplemented with 0.768 mg/L each of arginine, isoleucine, lysine, phenylalanine, and tyrosine; 1.152 mg/L aspartic acid; 0.256 mg/L methionine; 1.536 mg/L threonine; 2.176 mg/L valine; and 2% D-glucose. Depending on the auxotrophic markers required, SC medium was further supplemented with 0.025 mg/mL each of histidine, uracil, tryptophan, and adenine, and with 0.05 mg/mL leucine. Either *Saccharomyces cerevisiae* or *Escherichia coli* DH5α were used for strain construction. Yeast strains were generated using the standard lithium acetate (LiAc) transformation method. *E. coli* DH5α was routinely cultured at 37 °C in lysogeny broth (LB) medium with shaking at 250 rpm or on LB agar plates. DNA concentrations were measured using either a NanoDrop spectrophotometer (Thermo Fisher Scientific) or a Qubit fluorometer (Thermo Fisher Scientific). All construct assembly steps were verified by PCR, restriction enzyme digestion, or Sanger sequencing. The proofreading Phusion DNA polymerase (Thermo Fisher Scientific) was used for all PCR-based cloning.

Arrest and release of yeast cell cycle

Logarithmically growing cells were arrested in G1 phase by incubation in YPD medium supplemented with α-factor (3 μg/mL) for 2 h. Cell cycle arrest was confirmed by examining cell morphology. Following arrest, cells were washed three times with room-temperature YP medium (lacking glucose) and resuspended in fresh YPD medium to allow cell cycle re-entry. Cultures were incubated for 4 h to monitor recovery of normal cell cycle progression. Samples were collected periodically to assess cell morphology, size, and DNA content.

Fluorescence-activated cell sorting (FACS)

Cell sorting was performed using a FACSAria cytometer (BD Biosciences) with PBS as the sheath fluid. A 70-µm nozzle was used, and the sorting mode was set to "Purity." The flow cytometer voltage for Side Scatter (SSC) and Fluorescein Isothiocyanate (FITC) channels were set at 311 and 453, respectively. Forward Scatter (FSC) voltages were adjusted between 220 V and 400 V to optimize resolution across cell sizes.

The flow rate was set to $1\ \mu L \cdot s^{-1}$, and samples were diluted as needed to keep the event rate below $9{,}000\ events.\ s^{-1}$. Cultures were diluted as needed in 50 mM Tris-HCl (pH 7.5) and sonicated for 50 s at 14% amplitude using a Branson SLPe Digital Sonifier. Gating was performed on bivariate dot plots to identify single yeast cells. The initial gate was set on FITC-A (Area) versus FSC-H (Height) to select events with high fluorescence in the FITC channel, corresponding to the expression of the fluorescent reporter. Cells were then gated on SSC-A versus FSC-A. Doublets were excluded in two steps to prevent sorting of aggregated cells using FSC-W (Width) versus FSC-A, and FSC-H versus FSC-A gating. Cells were finally sorted based on FSC-A, used as a proxy for cell size. The smallest 7% of the population (corresponding to small-sized cells) was defined as the SMALL population. Each day, 100,000 cells from the lower 7% fraction were collected. Sorted cells were recovered in Eppendorf tubes containing 500 µL of YPD medium and subsequently transferred to glass tubes containing 10 mL of fresh YPD.

Clone selection by Forward Scatter (FSC-A)

Single colonies from each evolved population at 1,500 generations were picked from YPD plates and inoculated into liquid YPD medium, followed by overnight incubation at 30 °C. Cultures were then diluted and analyzed using an LSRFortessa flow cytometer (BD Biosciences). FSC-A was used as a proxy for cell size, while doublets and debris were excluded based on FSC-H versus FSC-A and SSC-A gating. For each clone, at least 10,000 singlet events were collected at a low flow rate. The median FSC-A value per clone was used for comparative analyses. Clones S1Cl2 and S2Cl4 were selected to proceed with the experiments requiring an isogenic background.

Evolution Experiments

The size selection experiment was initiated using the haploid ancestral strain yMF1001, grown overnight in rich YPD medium. At the start of the experiment, the ancestral culture was sorted by FACS to generate three distinct populations (S1-3 lineages). Cells were selected based on their small FSC-A values relative to the rest of the population, as previously described.

Populations were cultivated in glass tubes on roller drums at 30 °C and grown for 20 h. Each day, cultures were sorted, and 100,000 sorted cells were resuspended in 10 mL of fresh YPD medium,

allowing approximately 11–13 generations per cycle. Populations were propagated for a total of 116 cycles (≈ 1,500 generations). At each cycle, 800 µL of each evolving population was mixed with 800 µL of 30% (v/v) glycerol and stored at –80 °C for long-term preservation. The evolution experiment in the absence of size selection was performed using the Wild-Type (WT) strain, the ancestral strain, and two single clones isolated at the end of the size-selection based evolution experiment. In addition, a *sch9Δ* mutant was included. Each strain was inoculated into glass tubes containing 10 mL of YPD medium. After 24 h of growth, three independent replicates were established per strain, yielding a total of 12 evolving populations. Cultures were incubated under the same conditions as in the original evolution experiment, at 30 °C with continuous rotation, and grown for 24 h each cycle. Daily passages were performed by transferring 10 µL of the previous culture into 10 mL of fresh YPD (1:1000 dilution), allowing for approximately 10 generations per cycle. Populations were propagated for 15 cycles (≈ 150 generations). Every five cycles (≈ 50 generations), 800 µL of each population was mixed with 800 µL of 30% (v/v) glycerol and stored at –80 °C for long-term preservation.

Whole genome sequencing (WGS)

Genomic DNA was extracted by standard ethanol precipitation from 1 mL of yeast culture. Population samples collected at generations 333, 666, 1000, 1250, and 1500 were sequenced to identify genomic variants. Sequencing was also performed on populations derived from bulk segregant analysis.

Sequencing depths were approximately 25x for individual clones, 200x for population samples, and 500x for bulk segregant populations. Genomic DNA libraries were prepared following the protocol described by Koschwanez *et al.* (3) using the Nextera DNA Library Prep Kit (Illumina, RRID:SCR_010233; San Diego, CA, USA). DNA library concentration and quality were assessed with a GloMax® Plate Reader (Promega) and the Quant-iT™ PicoGreen® dsDNA Assay (Thermo Fisher Scientific). Paired-end sequencing was performed on an Illumina NextSeq 2000 platform.

Convergent evolution on genes

This method was performed based on the analysis of Fumasoni and Murray (4). Briefly, it relies on the assumption that those genes that have been mutated significantly more than expected by chance alone, represent cases of convergent evolution among independent lines. The mutations affecting those genes are therefore considered putatively adaptive. Per-base mutation rates were calculated as the number of mutations in coding regions divided by the total coding sequence length of the yeast genome in bp, for a given genetic background (including 1000 bp per ORF to account for regulatory regions).

$$\lambda = \frac{SNPs + indels}{\text{Coding Base Pairs}}$$

If the mutations were distributed randomly in the genome at a rate λ, the probability of finding n mutations in a given gene of length N is given by the Poisson distribution:

$$P(n\ mutations|gene\ of\ length\ N) = \frac{(\lambda N)^n e^{-\lambda N}}{n!}$$

For each gene of length N, we then calculated the probability of finding n mutations if these were occurring randomly:

$$P(\geq n\ mutations|gene\ of\ length\ N) = \sum_{k=n}^{\infty} \frac{(\lambda N)^n e^{-\lambda N}}{n!} = 1 - \frac{\Gamma(n, \lambda N)}{(n-1)!}$$

(where Γ is the upper incomplete gamma function) which gives us the P value for the comparison of the observed mutations with the null, Poisson model. To decrease the number of false positives, we then performed multiple-comparison corrections by using the Bonferroni correction. The custom pipeline used for the data analysis is available on GitHub (https://github.com/FumaLab/Garona2025).

Bulk segregant analysis

Bulk segregant analysis (BSA) is an experimental approach used to identify genetic loci associated with a specific phenotype (3, 5). In this study, BSA was performed by crossing a clone from the evolved population exhibiting the phenotype of interest with a WT strain lacking that phenotype. The resulting diploids were sporulated, allowing random segregation of the mutations present in the evolved clone. Haploid segregants were selected for both growth and the phenotype of interest—in this case, small cell size—over approximately 40–50 generations by sorting as described for the evolution experiment set-up. The fittest cells carrying putative mutations became enriched in the population during selection. Segregants displaying the target phenotype were subsequently pooled, and their genomic DNA was extracted, sequenced, and analyzed to identify mutations associated with the phenotype. BSA was performed as follows. One clone from each evolved population was selected as described above (see *Clone selection by Forward Scatter*) and mated with a haploid WT strain of the opposite mating-type (yMF0473: *MATa ade2-1; can1-100; leu2-3,112; his3-11,15; ura3-1; trp1-1; RAD5⁺; pSTE2-URA3::KanMX4*). In this WT, the native *URA3* promoter was replaced with the *STE2* promoter, which is active only in MATa cells, allowing for the selection of MATa spores after meiosis since neither MATα haploids nor MATa/MATα diploids can express *URA3* under the *STE2* promoter.

Mating was carried out on YPD plates by mixing cells from both strains. The crosses were incubated overnight at 30 °C, after which cells were scraped from the plates and grown in double-selective synthetic medium lacking histidine and tryptophan (–HIS –TRP) to select for diploids. Diploid cultures (100 mL) were grown to saturation, centrifuged, and resuspended in synthetic medium containing

2% potassium acetate (KAc) to induce sporulation. Cultures were incubated overnight (12–15 h) at 30 °C, pelleted, and resuspended in water containing 2% KAc. Sporulation was continued for 7 days at room temperature (23–25 °C) with agitation on a roller drum. Sporulation efficiency was verified microscopically and was approximately 15%, yielding 0.5–1 × 10^9 spores. To digest asci, 100 mL of the sporulated culture was pelleted and resuspended in 5 mL of water containing 2500 units of Zymolyase (Zymo Research). The suspension was incubated with shaking at 30 °C for 1 h, with intermittent vortexing. When most tetrads were separated, 40 mL of water and 5 mL of 10% Triton X-100 were added, followed by 1 min of sonication at 10-15% amplitude to disperse remaining aggregates. Spores were collected by centrifugation (6000 rpm, 1 min) and resuspended in haploid selective medium lacking uracil (–URA). After 48 h of growth, spores were subjected to Fluorescence-Activated Cell Sorting to select for cells with small size and high fitness, as previously described. This selection regime, analogous to that used during the experimental evolution, enriches for cells displaying the target phenotype. After three rounds of selection, genomic DNA was extracted from the final saturated cultures and used for library preparation and high coverage WGS, as mentioned above. A custom pipeline was used to identify alleles segregating with the evolved phenotype. Variants present at frequencies greater than 50% were considered non-randomly segregating, consistent with inheritance from the evolved parent in the diploid cross (haploid evolved × haploid WT). A second BSA was performed with clones isolated from the first one to narrow down the set of candidate mutations: the first bulk yielded approximately 30–80 segregating mutations, whereas the second bulk reduced this number to around 10 candidate mutations.

Fitness assays

The relative fitness (ω) of WT, ancestral, evolved, or constructed strains relative to a WT reference strain was determined through direct pairwise competition experiments, each performed in triplicate. The reference strain was haploid *S. cerevisiae* W303 (yMF1176: *can1; ADE2; leu2-3,112; his3-11,15; ura3-1; trp1-1; RAD5$^+$; yel068c::CAN1/URA3; prACT-Citrin-LEU2; prACT-Cerulean-KANMX*). All strains were preconditioned by growth to saturation in liquid YPD medium prior to competition. Competing strains were mixed at defined initial ratios (typically 1:1 or 20:80 depending on competing strain growth) and incubated at 30 °C for 24 h. Cultures were serially transferred through 1:1000 dilutions for three consecutive 24 h cycles, and the relative abundances of competing strains were quantified at each transfer. The reference strain expressed a Cerulean fluorescent protein, which was absent in the WT, ancestral, evolved, and constructed strains, allowing strain discrimination. Population sizes were determined based on the number of events detected by flow cytometry (LSRFortessa, BD Biosciences). Relative fitness was estimated from changes in ratio of competing strains over time.

Specifically, strain ratios (r) were calculated from the number of cerulean-positive ($\mathrm{N_C}$) and cerulean-negative ($\mathrm{N_{CN}}$) events measured by flow cytometry:

$$r = \frac{N_{Cerulean-Negative}}{N_{Cerulean}}$$

The number of generations (g) between time points was estimated from total cell counts measured at 0 h and 24 h:

$$g = \frac{\ln(N_t/N_0)}{\ln(2)}$$

Relative fitness (ω) was then calculated as the slope of the linear regression between $\ln(r)$ and the number of generations (g) for each competition experiment:

$$\omega = \frac{\Delta \ln(r)}{\Delta g}$$

Relative fitness values were normalized to those of the WT or ancestral strains, depending on the comparison. Mean relative fitness values were obtained from at least three independent biological replicates. Reported P-values were derived from two-tailed t-tests. The custom data analysis pipeline is available on GitHub (https://github.com/FumaLab/Garona2025).

Cell cycle profiles

Cell cycle analysis was performed on cells collected from exponentially growing cultures. Cells were harvested by centrifugation (2 min at 6000 rpm) and resuspended in 70% ethanol containing 250 mM Tris-HCl (pH 7.6). The suspension was sonicated for 10s at 10% amplitude using a Branson SLPe Digital Sonifier and incubated for 1 h at room temperature. Cells were then washed with 50 mM Tris-HCl (pH 7.5), resuspended in the same buffer supplemented with 0.04 mg/mL RNase A (Sigma-Aldrich), and incubated at 37 °C for at least 1 h. Following RNase treatment, cells were pelleted and resuspended in 50 mM Tris-HCl (pH 7.5) containing 0.2 mg/mL proteinase K (GRiSP), and incubated for 30 min at 50 °C. After centrifugation, cells were resuspended in 50 mM Tris-HCl (pH 7.5). Samples were diluted 10–20-fold in 50 mM Tris-HCl (pH 7.5) containing 1 mM SYTOX Green and sonicated again for 10 s at 10% amplitude. Stained samples were analyzed on an LSRFortessa flow cytometer (BD Biosciences) using the FITC channel to quantify cellular DNA content. Flow cytometer settings were as follows:

- 10,000 events acquired per sample; FSC 340 V; SSC 200 V; FSC-PMT (Photomultiplier Tube) 130 V; SSC(BV) (Brilliant Violet) 190 V; and FITC 535–590 V.

Cell cycle profiles were analyzed and visualized using a custom Python script based on the *FlowCytometryTools* package (https://pypi.org/project/FlowCytometryTools/). The fraction of the doubling time spent by each sample in each cell cycle phase was calculated using RepliFlow (6).

Growth curves

Overnight cultures were diluted 1:300 (50 µL into 15 mL of total volume) into rich medium and grown at 30 °C in culture tubes with continuous shaking to minimize sedimentation. Cell density was measured every hour with a Coulter Counter (Multisizer 4e, Beckman Coulter). Time-series data were analyzed in Python v3.9 using a custom script based on growth_curve_analysis.py (https://github.com/nwespe/OD_growth_finder), and the "effective growth rate" mode was used to calculate growth rates.

Mating type switch

Clones from evolved populations S1 and S3 were transformed with a *pGAL1-HO* (HO: Homothallic switching endonuclease) plasmid conferring nourseothricin resistance. Evolved clones from population S2 failed to switch mating type. Single transformant colonies were grown for 24 h at 30°C in liquid YP medium supplemented with 2% raffinose to relieve the repression of the *GAL1* promoter. The following day, cultures were diluted 1:10, and galactose was added to a final concentration of 2% to induce expression of HO endonuclease. After 90 min of induction to allow for mating-type switching, cultures were diluted 1:100 and plated onto YPD plates. Successful mating-type switches were verified by mating the resulting cells with tester strains of known mating type.

Volumetric measurements

Cell volume was determined using a Coulter Counter (Multisizer 4e, Beckman Coulter), which measures particle size based on the Coulter principle—each cell passing through an electrolyte-filled aperture generates an impedance change proportional to its volume (7). Before measuring, cultures were sampled (500 µL) and briefly sonicated in a 1.5 ml microcentrifuge tube, for 10–30 s at 10% amplitude using a Branson SLPe Digital Sonifier to reduce cell clumping. Cells were diluted in 25 ml vials (Beckman Coulter Accuvette ST 25 ml) containing 10ml of Coulter® ISOTON® II Diluent solution. For exponential phase measurements, cells were sampled 7h after a 1:200 dilution, and 100 µL of sonicated culture were used. For stationary phase measurements, cells were collected 48h after the dilution, with 10 µL of sonicated culture taken for analysis. Measurements were all performed using a 50 µm aperture tube. For all volume estimations, a minimum of 30,000 events were recorded, and particles smaller than 1.5 fL or larger than 2500 fL were excluded from analysis. Raw data are available at Zenodo (8). Data analysis was performed using a custom Python pipeline available on GitHub (https://github.com/FumaLab/Garona2025).

Quantification of modal volumes and of the width of cell volume distributions

Cell size measurements obtained with the Coulter Counter included noise contributions from debris and non-cellular particles. Such artifacts affect calculations of summary statistics such as the mean,

median, mode, and standard deviation (SD). Therefore, we characterized each cell size distribution by two statistics robust to low-volume debris: the modal volume and the normalized mean excess above the mode.

The modal volume v_{mode} was estimated as the location of the maximum of a Kernel Density Estimate (KDE) of the cell volume distribution, restricted to volumes above a strain-specific debris cutoff (Fig. 4B). Because debris particles are smaller than intact cells, the mode of the cellular peak is unaffected by their presence as long as they are well separated from the main distribution.

To quantify the width of the right tail of the size distribution, we computed the mean excess above the mode, defined as $M = E[v - v_{mode} | v \geq v_{mode}]$.

This quantity measures the average distance of cells at or above the mode from the modal volume, i.e. the average "extra size" carried by cells in the right tail. Normalizing by the modal volume yields the dimensionless ratio $nME = M/v_{mode}$ reported in Fig. 4B. This metric is a natural generalization of the right Half-Width at Half Maximum (HWHM) of the size distribution. The HWHM measures the distance from the mode to the point where the density falls to half its peak value: a single horizontal slice of the distribution. By contrast, it can be understood as the average of all such right half-widths across every density level h from 0 to $p(v_{mode})$: by the layer-cake identity,

$$\int_{v_{mode}}^{\infty} (v - v_{mode}) p(v) dv = \int_{0}^{p(v_{mode})} w(h) dh,$$

$$\text{where } w(h) = sup\{v \geq v_{mode} : p(v) \geq h\} - v_{mode}$$

is the right semi-width of the distribution at height h (Fig. 4C, right panel). Thus, M captures the full shape of the right tail rather than its width at one arbitrary height, while remaining interpretable in the same units as the HWHM.

RNA extraction

Total RNA was extracted from yeast cultures with three biological replicates per strain. 1.5×10^7 cells were collected during exponential growth phase. RNA was extracted with the YeaStar™ RNA Kit from Zymo Research. RNA integrity was evaluated by the RQN parameter (RNA Quality Number) with the 5200 Fragment Analyzer (Agilent Technologies) according to the manufacturer's instructions. RQN was > 6 in all the samples. RNA concentration was measured with the NanoDrop spectrophotometer (Thermo Fisher Scientific).

Protein extraction

Total protein was extracted from yeast cultures with three biological replicates per strain. 1×10^8 cells were collected during exponential growth phase. Proteins were extracted with a Trichloroacetic

Acid (TCA) based method. Cells were harvested by centrifugation for 3 min at 4,000 rpm. The supernatant was discarded, and cell pellets were resuspended in 2 mL of 20% TCA in 2 mL Eppendorf tubes. Cells were then centrifuged for 1 min at 13,000 rpm, and pellets were resuspended in 200 µL of 20% TCA. Glass beads were added to the suspension, leaving a small volume without beads, and samples were vortexed at 4°C for 3 min to lyse the cells. Subsequently, 400 µL of 5% TCA was added, and the mixture was centrifuged for 10 min at 3,000 rpm. The supernatant was carefully transferred to a fresh 1.5 mL Eppendorf tube. Pellets were allowed to dry completely on paper. Dried pellets were resuspended in 100 µL of Bicinchoninic Acid (BCA) assay-compatible extraction buffer (50 mM Tris-HCl, pH ~7.5; 150 mM NaCl; 0.5% non-ionic detergent such as Triton X-100 or NP-40/IGEPAL) for downstream protein quantification. Protein concentration was determined using a BCA assay according to the manufacturer’s instructions.

Electron microscopy (EM)

Saccharomyces cerevisiae cells were grown in YP medium supplemented with 0.5% D-glucose. This reduced glucose concentration was chosen according to yeast EM best practices to improve cell wall permeability. A total of 10–15 OD_{600} units of cells were collected and centrifuged at 1000 g (≈3000 rpm) for 3–5 min at room temperature. The resulting pellet was resuspended and washed for 10 min with 10 mL of distilled water. This washing step is essential for autophagic body analysis, as it promotes the formation of a single large vacuole. Cells were then resuspended in 1.5 mL of freshly prepared ice-cold 1.5% $KMnO_4$ fixative and transferred into 1.5 mL microcentrifuge tubes. Tubes were filled with the fixative to eliminate air and incubated on a rotating wheel for 30 min at 4 °C. Cells were centrifuged at 1400 × g (≈4000 rpm) for 3 min at 4 °C, resuspended in 1.5 mL of ice-cold 1.5% $KMnO_4$, and incubated overnight on a rotating wheel at 4 °C. The following day, cells were washed five times with distilled water, centrifuging each time at 2200 × g (≈5000 rpm) for 3 min. Samples were then dehydrated through a graded acetone series (10%, 30%, 50%, 70%, 90%, 95%, and 100%), with each step lasting 20 min. Between each step, cells were centrifuged at 2200 × g (≈5000 rpm) for 4 min.

After dehydration, samples were infiltrated with SPURR resin. The infiltration was performed sequentially as follows: (1) 1 part resin to 3 parts acetone for 1 h at room temperature; (2) 1:1 resin-to-acetone for 1 h; and (3) 3:1 resin-to-acetone for 1 h, with centrifugation (≈5000 rpm, 3 min) between each step. Finally, samples were incubated overnight in 100% resin. The following day, samples were centrifuged at 2200 × g (≈5000 rpm) for 5 min, the supernatant discarded, and a final infiltration in 100% resin was performed for 1 h at room temperature on a rotating wheel. Samples were transferred to conical embedding capsules, centrifuged (5000–7000 rpm, 3–5 min), the supernatant discarded, and the tubes filled with fresh 100% SPURR resin. Polymerization was carried out at 60–70 °C for at least 3 days. Polymerized samples were sectioned to a depth of approximately 10 µm using standard methods, and ultrathin sections (≈80 nm thick) were obtained

with a diamond knife. Sections were imaged on a Tecnai G2 Spirit BioTWIN transmission electron microscope (FEI) equipped with an Olympus-SIS Veleta CCD camera. Three-dimensional image reconstruction was performed using Amira software (Thermo Fisher Scientific). Image stacks were aligned, and organelles were manually segmented and identified.

Distribution of cell volumes across the *Saccharomycotina* subphylum

Cell volumes were estimated from the reported mean cell length and width (μm) from (9) by approximating each morphology with an idealized geometric solid. For each annotated shape class, a corresponding analytical formula (e.g., sphere, ellipsoid, cylinder, spherocylinder, cone, or frustum) was used to compute volume. When multiple shape annotations were present for a given cell type, volumes were calculated independently for each applicable geometry and averaged to obtain a single estimate. Invalid or non-physical measurements (e.g., negative or non-finite values) were excluded. All volumes were expressed in femtoliters, using the identity 1 μm^3 = 1 fL. The code used for these calculations is available in a public GitHub repository. (https://github.com/FumaLab/Garona2025).

Figures

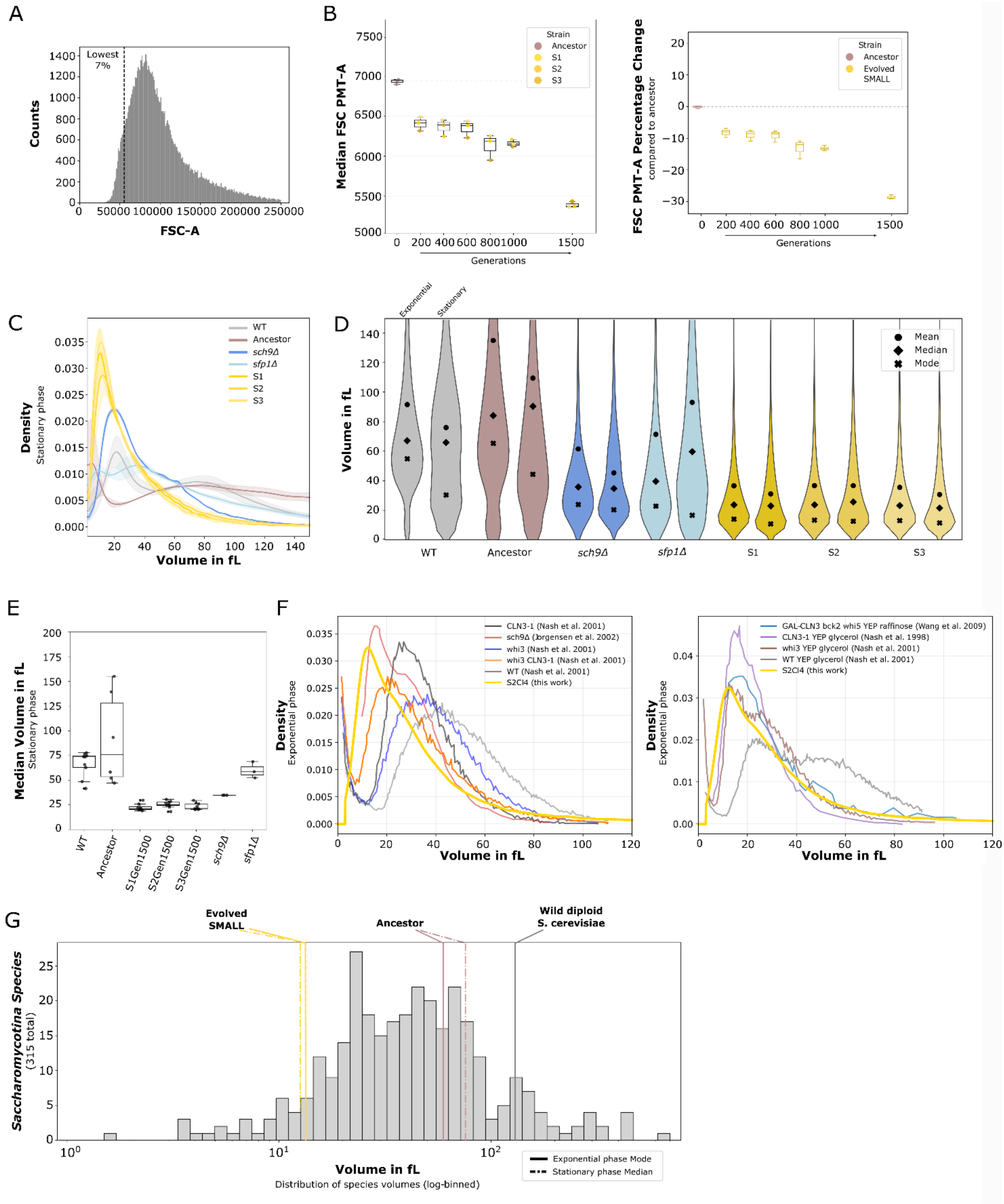

Fig. S1. (A) Selection of the lowest 7% of the population based on FSC-A. (B) Changes in median (left) and relative (right) FSC PMT-A during experimental evolution across populations. (C) Cell volume distributions in stationary-phase cultures. Smooth density curves represent kernel density estimates fitted to histograms of cell volumes. For each strain, densities were computed per replicate and averaged to obtain a mean density curve. Shaded areas represent the standard deviation across replicates. (D) Cell size dynamics across growth

phases. Violin plots show the average distribution of cell volumes (fL) measured in exponential and stationary phases. Summary statistics highlighted were derived from all individual measurements. (E) Median cell volume from measurements of stationary -phase cultures (Coulter counter). Box plots show medians, interquartile ranges, and whiskers (1.5× IQR). (F) Cell volume distributions for the evolved clone S2Cl4 and previously reported small-size mutants were measured under standardized conditions. Left: Cells grown in rich medium (YPD) and sampled during exponential phase. Right: Cells grown in nutrient-limiting media containing poor carbon sources (e.g., glycerol or raffinose) and measured during exponential phase. Cell size distributions for reference strains were digitized from published figures (10–13). (G) Distribution of cell volumes across the *Saccharomycotina* subphylum, with volumes estimated from cell diameters and shapes reported in (9). Only 29 of 315 species (9.21%) have a volume ≤14 fL, comparable to the evolved SMALL strain.

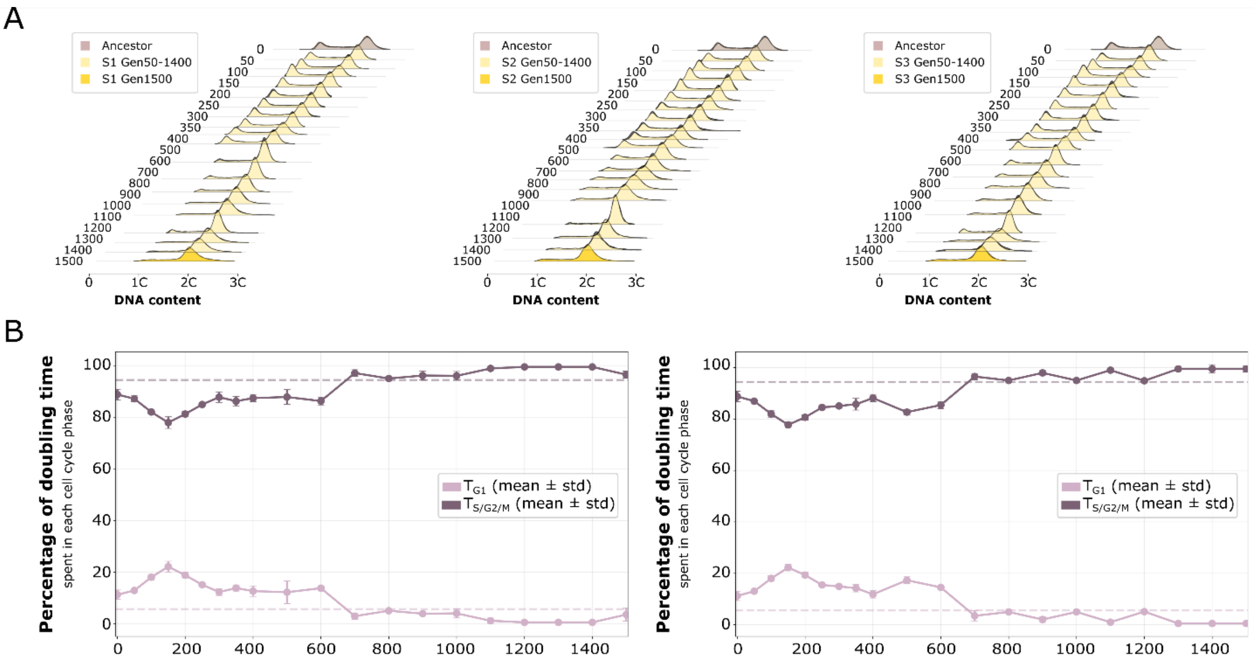

C

Type	Cell / Nucleus	MTC / Nucleus	Vacuole / Nucleus
WT1	10,89	0,44	0,78
WT2	13,11	0,30	1,33
WT3	11,48		
WT4	12,54	0,45	1,48
WT5	11,41	0,42	0,86
SMALL1	10,78	0,32	0,28
SMALL2	7,66	0,41	0,29
SMALL3	7,97	0,33	0,23
SMALL4	9,06	0,29	0,62
SMALL5	9,38	0,33	0,71
**Average**			
WT	11,89	0,40	1,11
SMALL	8,97	0,34	0,43

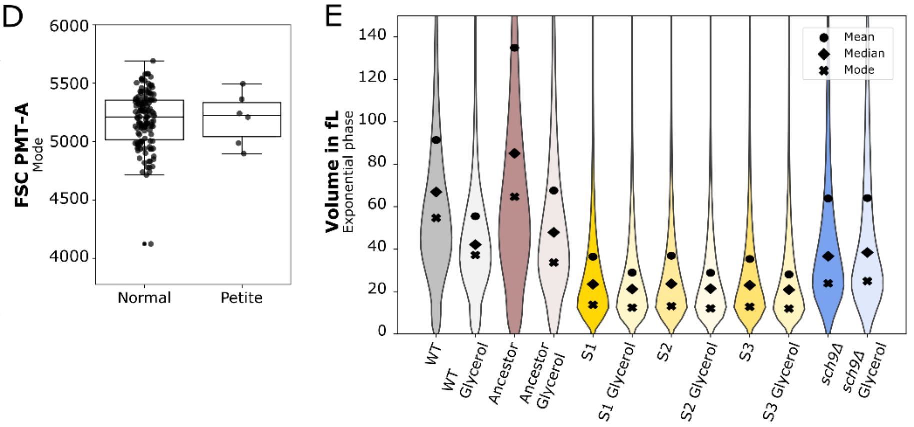

Fig. S2. (A) Cell cycle profiles of SMALL lineages S1–S3, shown without normalization to the G2 peak. DNA content was estimated using SYTOX Green staining (~10,000 cells per histogram). The x-axis shows arbitrary fluorescence units; the y-axis, relative cell frequencies. Solid lines denote mean profiles; shaded areas, SD (n = 3). Peaks for 1C (G1) and 2C (G2/M) DNA content are indicated. (B) Allocation of time to G1 and S/G2/M phases in S2 (left) and S3 (right) populations, inferred from DNA content profiles. Phase durations were calculated following (6). Bars show mean ± SD (n = 3). (C) Transmission electron microscopy (TEM) measurements of cell and organelle size, with nuclear size included as an internal control. The WT3 sample was excluded due to a sectioning artifact during sample preparation that compromised the nucleus area. (D) Mitochondrial functionality assessment. Among 137 isolated clones, 6 (4.3%) displayed mitochondrial defects. The y-axis shows FSC-PMT-A values indicating size similarity between petites and normal cells. (E) Cell size across carbon sources. Violin plots show the average distribution of cell volumes (fL) measured in rich media with glucose or glycerol as a carbon source. Summary statistics highlighted were derived from all individual measurements.

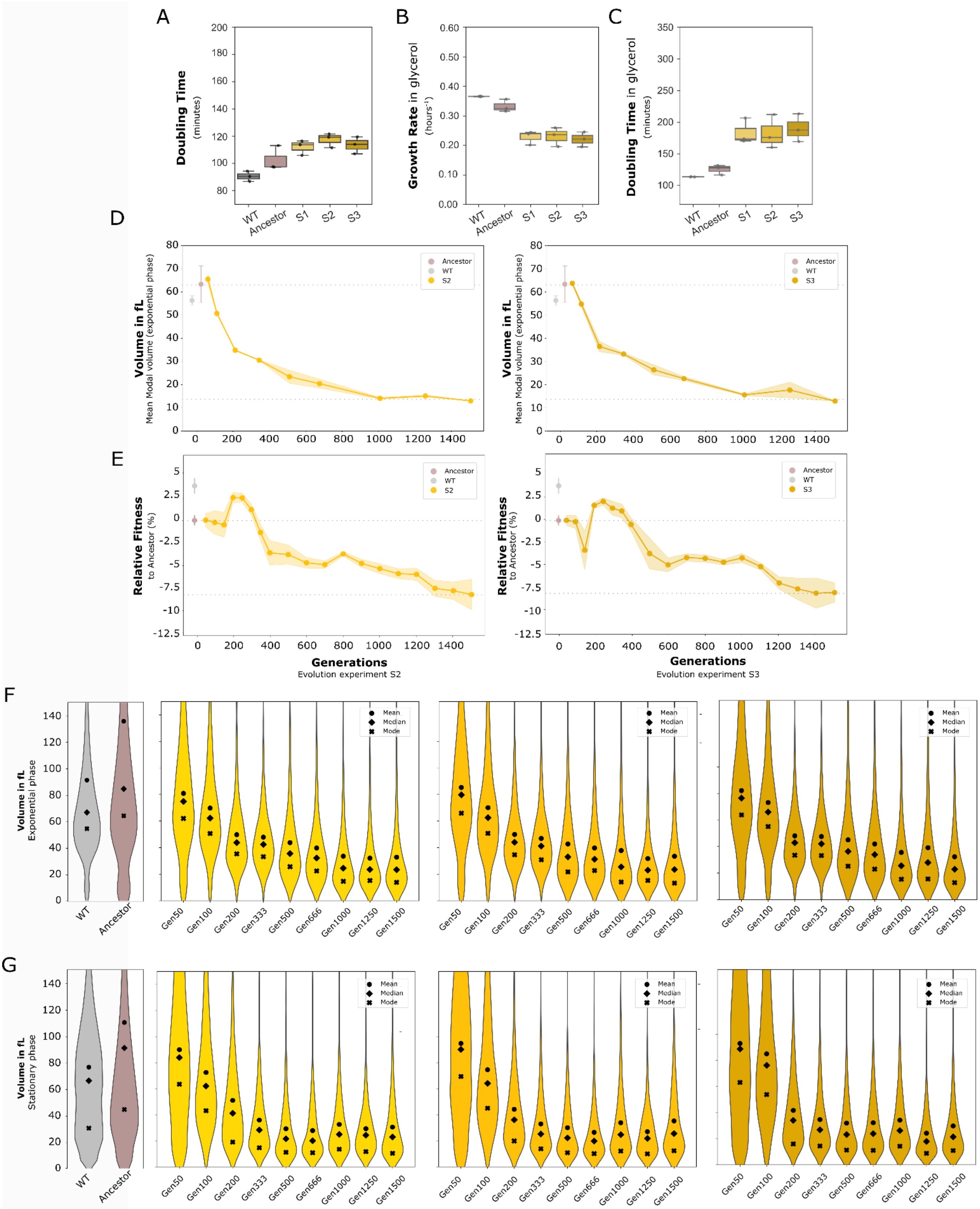

Fig. S3. (A) Doubling times of reference and evolved strains (generation 1,500) in YPD. (B) Growth rates of reference and evolved strains in rich medium with glycerol as a carbon source. (C) Doubling times of reference and evolved strains in glycerol medium. Box plots show medians, interquartile ranges, and whiskers (1.5× IQR). (D) Size trajectories over 1,500 generations. Solid lines represent the mean mode, and shaded areas

indicate SD. (E) Trajectories of fitness relative to the ancestor over 1,500 generations. Solid lines represent mean fitness, and shaded areas indicate SD. (F–G) Cell size trajectories over 1,500 generations. Violin plots show average cell size distributions in exponential phase (F) and stationary phase (G). The first panel includes the WT and ancestor as references, followed by distributions for the three evolving populations across generations. Summary statistics highlighted were derived from all individual measurements.

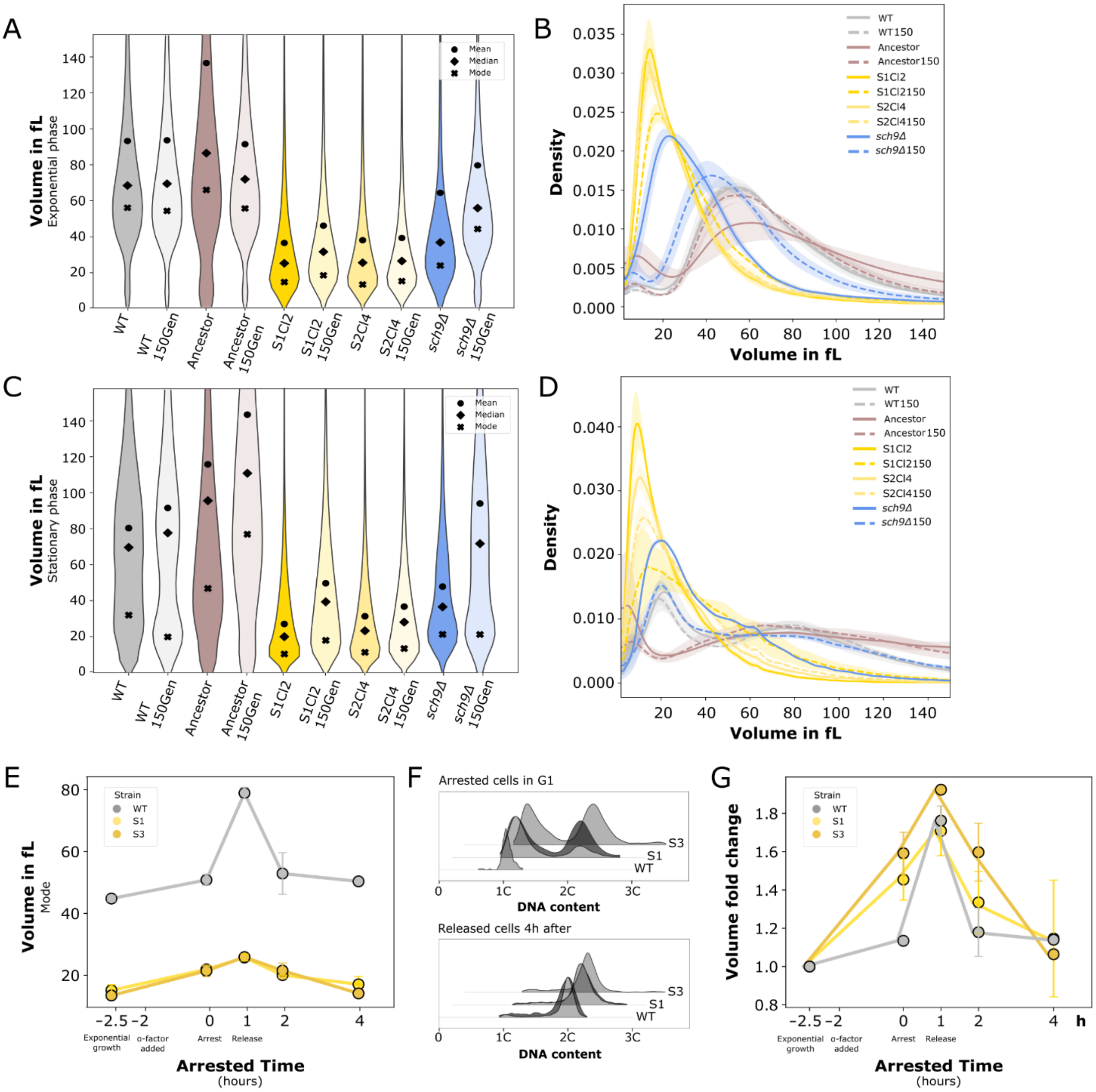

Fig. S4. (A–D) Cell size distributions before and after 150 generations of evolution without size selection. Violin plots show average cell volumes (fL) in exponential-phase (A) and stationary-phase (C), while density plots show kernel density estimates of the same measurements in exponential-phase (B) and stationary-phase (D). For each strain, densities were computed per replicate and averaged to obtain a mean density curve, and shaded areas represent the standard deviation across replicates. Summary statistics highlighted were derived from all individual measurements. (E) Cell volume dynamics during synchronization with α-factor. Upon G1 arrest, cells maintained larger volumes, followed by a progressive decrease after release into fresh medium. (F) Cell cycle profiles from clones belonging to evolved pop S1 and S3: DNA content was quantified by SYTOX Green staining during exponential growth, α-factor arrest, and post-release. Arrested populations (above) accumulated with a 1C DNA peak (G1), while released cells (below) synchronously progressed through S-phase and restored a 2C peak (G2/M). Evolved strains showed less efficient arrest, with a fraction remaining in G2/M. Genome sequencing revealed mutations in pheromone signaling genes, potentially explaining reduced responsiveness to α-factor. The x-axis shows arbitrary fluorescence units, the y-axis, relative frequencies. (G) Fold change in cell volume relative to the mean exponential volume before arrest.

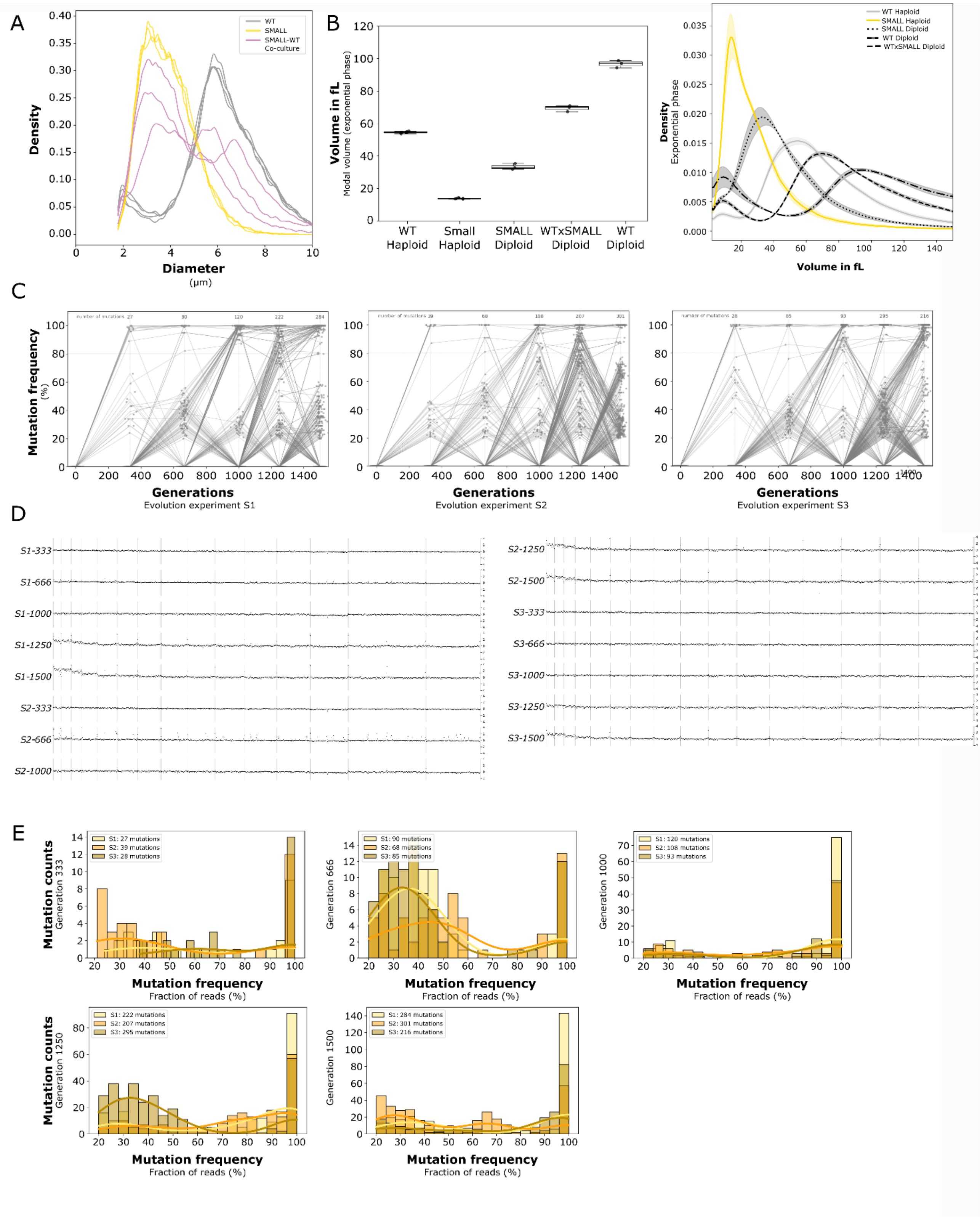

Fig. S5. (A) Coexistence of WT and evolved Small-cell strains during 72 h co-culture. WT and Small strains were mixed 1:1 and grown together for 48 hours. Cell size distributions were measured at stationary phase using a Coulter Counter (n = 3). No significant shifts in population size distributions were observed. (B) Haploid and diploid strains' modal volumes (left). Diploids were generated from crosses of Small × Small, Small × WT, and WT × WT strains. Density plots (right) show cell distributions for diploids generated from crosses. For each strain, densities were computed per replicate and averaged to obtain a mean density curve. Shaded areas

represent the standard deviation across replicates. (C) Allele frequency trajectories of mutated genes in Small populations S1–S3. (D) Copy number profiles of linear chromosomes across sequenced populations during experimental evolution (generations 333, 666, 1,000, 1,250, and 1,500). At 1,250 and 1,500 generations, apparent increases in small chromosome copy number are artifacts of DNA extraction bias, likely due to partial cell wall digestion that preferentially releases small chromosomes. (E) Distribution of mutated read fractions across Small populations. The mutation fraction (%) was calculated as the proportion of sequencing reads containing a given mutation. Histograms are overlaid with kernel density estimates (KDE, colored lines). Read fraction distributions were compared using Kolmogorov–Smirnov (KS) tests (full statistics in Dataset S7).

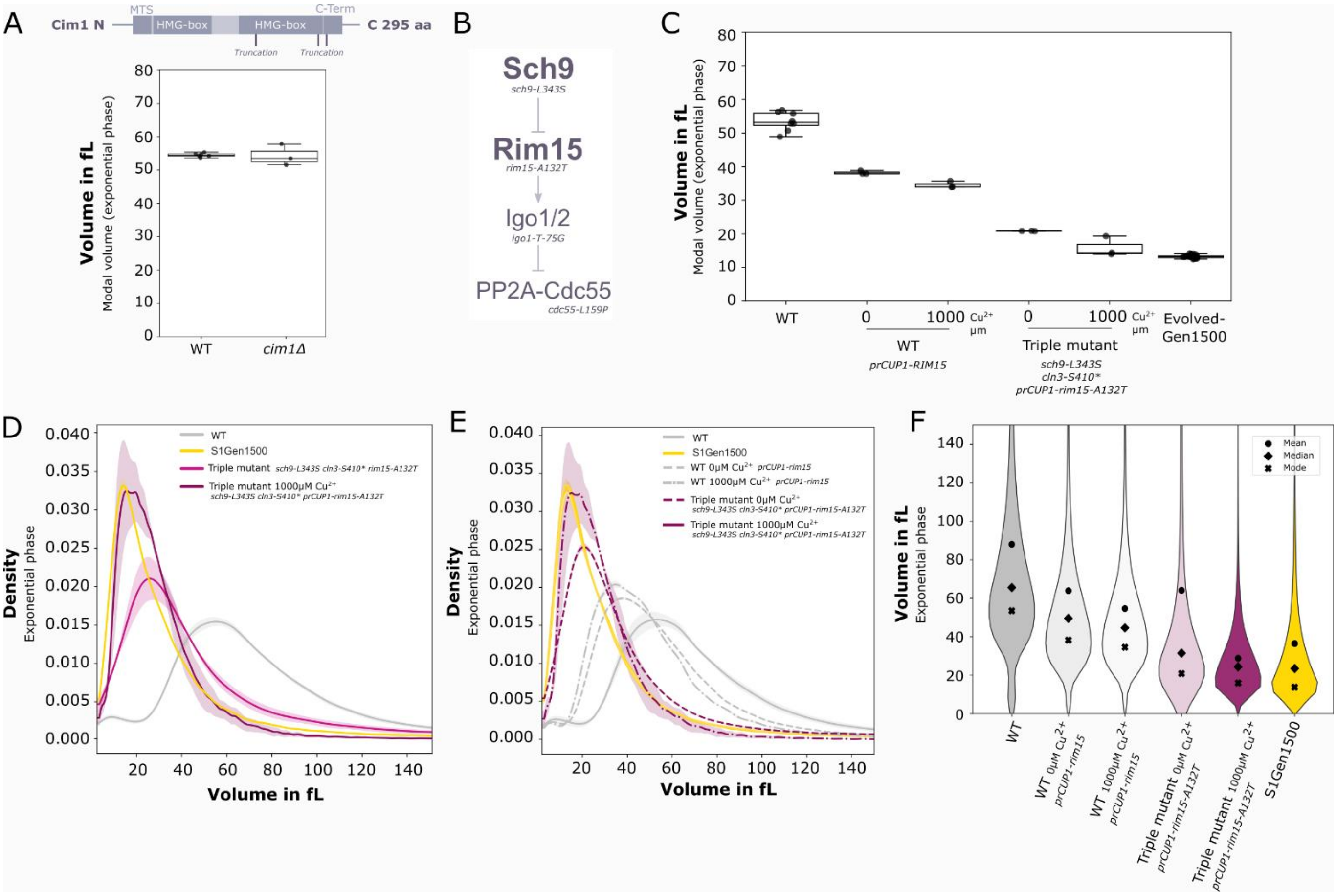

Fig. S6. (A) Above: Schematic representation of mutations identified in the *CIM1* gene, with affected domains indicated (adapted from (14)). Below: Volumetric measurements of *cim1Δ* strain, compared to WT. Box plots show medians, interquartile ranges, and whiskers (1.5× IQR). (B) Schematic representation of the Greatwall kinase pathway with experimentally identified mutations. (C) Cell volumes measured in exponential-phase cultures (Coulter counter). Box plots as in panel A. Response of WT and triple mutant strains to increasing copper concentrations (0 and 1000 μM $CuSO_4$) is shown; prCUP1 denotes expression of RIM15 under control of the copper-inducible promoter. (D-E) Density distributions of cell volumes for the analyzed strains in exponential phase. For each strain, densities were computed per replicate and averaged to obtain a mean density curve, and shaded areas represent the standard deviation across replicates. (F) Violin plots showing average cell distributions in exponential phase. Summary statistics highlighted were derived from all individual measurements.

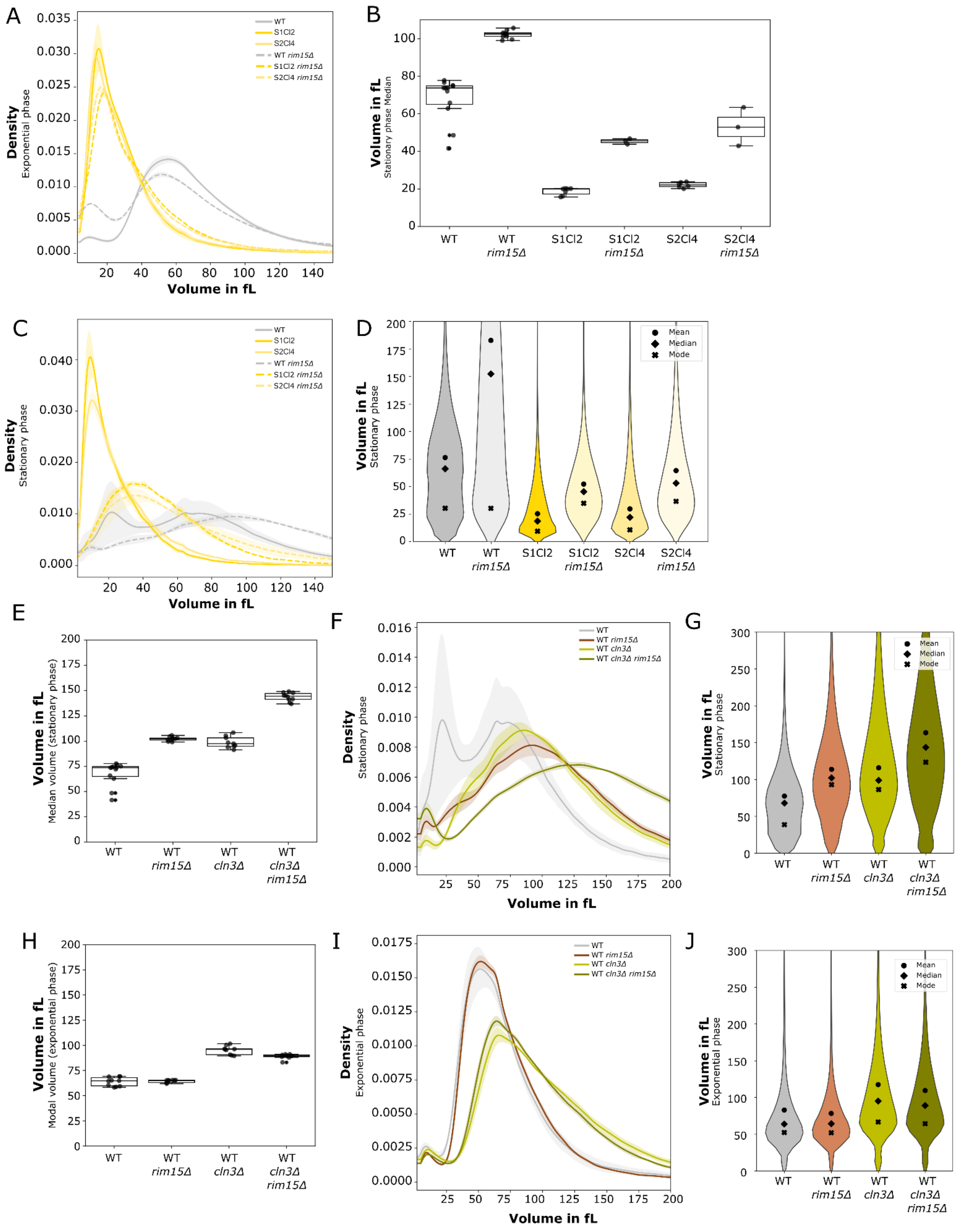

Fig. S7. (A) Density plots showing cell size distributions of *RIM15* deletions in exponential phase. (B) Median cell volumes and distributions for *RIM15* deletions in stationary phase. (C) Average density distributions and violin plots (D) for *RIM15* deletions in stationary phase. (E-G) Stationary-phase measurements for *rim15Δ*,

cln3Δ, and *rim15Δ cln3Δ* double mutant: box plots of median cell volumes (E), density distributions (F), and violin plots (G). (H-J) Exponential-phase measurements for *rim15Δ*, *cln3Δ*, and *rim15Δ cln3Δ* double mutant: box plots of modal cell volumes (H), average density distributions (I), and violin plots (J). Samples were sonicated (5 × 1 s pulses at 10% amplitude) prior to measurement. Density distributions were computed per replicate and averaged to obtain a mean density curve. Shaded areas represent the standard deviation across replicates. Summary statistics highlighted in violin plots were derived from all individual measurements. Box plots show medians, interquartile ranges, and whiskers (1.5 × IQR).

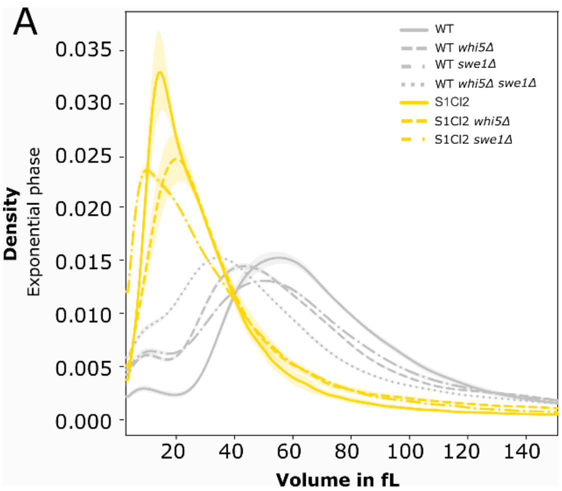

Fig. S8. (A) Density distributions of cell volumes for *swe1Δ* and *whi5Δ* mutants in exponentially growing cultures. For each strain, densities were computed per replicate and averaged to obtain a mean density curve. Shaded areas represent the standard deviation across replicates.

Dataset S1. Yeast strains used in this study.

List of *S. cerevisiae* strains employed in this work, including strain names, genotypes, and sources or construction details.

Dataset S2. Mutations identified in evolved lineages.

List of mutations identified in evolved yeast lineages. Each entry includes the gene name, SGD identifier, chromosome number, mutation type and position, and the subpopulation lineage in which the mutation occurred. Additional columns indicate the generation of detection, reference and mutant read counts, total reads, and the allele frequency (fraction).

Dataset S3. Genes previously implicated in cell size regulation.

List of genes identified in evolved yeast lineages that are reported in the literature to affect cell size in *S. cerevisiae*. The full list was obtained from the Saccharomyces Genome Database (SGD, https://www.yeastgenome.org/). Each entry provides a gene name, systematic name, associated phenotype, experimental approach, and experiment type category. Additional columns summarize mutant information, strain background, specific experimental details, and the corresponding reference.

Dataset S4. Genes under parallel evolution.

List of genes identified through statistical analysis as mutated more often than expected given their coding sequence length and the observed mutation rates, indicative of parallel evolution. Each entry includes the systematic name, SGD identifier, gene name, gene size, number of independent mutation hits, and counts of mutations in coding (CDS), noncoding (non-CDS) sequences, or synonymous mutations. Additional columns provide mutation frequency, zygosity, origin, gene description, and significance values (P and Bonferroni-corrected P (Pe)).

Dataset S5. Mutations identified by bulk segregant analysis.

List of mutations detected after two consecutive rounds of backcrossing and selection for small cell size, with corresponding SGD identifiers for each mutation.

Dataset S6. Summary of genes identified across analyses.

List of genes identified as putative adaptive candidates across the four complementary analyses (Tables S2–S5). Each entry includes the systematic name, counts and indicators used for ranking; SGD identifier, gene name, gene size, origin, and functional description.

Dataset S7. Raw data utilized to produce plots

Data Availability

The genomic datasets used within this publication were deposited at European Nucleotide Archive (PRJEB101156). Scripts used for data analysis are available at the GitHub repository (https://github.com/FumaLab/Garona2025). The raw data corresponding to each figure are provided in the Dataset S7. Expanded view data, supplementary information, and appendices are available for this paper. Raw data from Coulter Counter measurements is available at Zenodo (8).

SI References

1. Y. H. Jin, *et al.*, The multiple biological roles of the 3’-->5’ exonuclease of Saccharomyces cerevisiae DNA polymerase delta require switching between the polymerase and exonuclease domains. *Mol. Cell. Biol.* **25**, 461–471 (2005).
2. *Guide to yeast genetics and molecular biology* (Academic Press, 1990).
3. J. H. Koschwanez, K. R. Foster, A. W. Murray, Improved use of a public good selects for the evolution of undifferentiated multicellularity. *Elife* **2013**, e00367 (2013).
4. M. Fumasoni, A. W. Murray, The evolutionary plasticity of chromosome metabolism allows adaptation to constitutive DNA replication stress. *Elife* **9**, 1–28 (2020).
5. A. V. Segrè, A. W. Murray, J.-Y. Leu, High-resolution mutation mapping reveals parallel experimental evolution in yeast. *PLoS Biol.* **4**, e256 (2006).
6. A. Alsina, M. Fumasoni, P. Sartori, Model-based inference of cell cycle dynamics captures alterations of the DNA replication programme. *PLoS Comput. Biol.* **21**, e1013570 (2025).
7. W. H. Coulter, Means for counting particles suspended in a fluid. *US Patent* (1953).
8. A. Garoña, M. Fumasoni, A. Giometto, M. Lemos, Dataset_raw_coulter_counter files_Experimental evolution of cellular miniaturization suggests a mechanism for cell size evolution. Zenodo. https://doi.org/10.5281/ZENODO.19254220. Deposited 2026.
9. C. M. Chavez, *et al.*, The cell morphological diversity of Saccharomycotina yeasts. *FEMS Yeast Res.* **24**, foad055 (2024).
10. R. Nash, G. Tokiwa, S. Anand, K. Erickson, A. B. Futcher, The WHI1+ gene of Saccharomyces cerevisiae tethers cell division to cell size and is a cyclin homolog. *EMBO J.* **7**, 4335–4346 (1988).
11. R. S. Nash, T. Volpe, B. Futcher, Isolation and characterization of WHI3, a size-control gene of Saccharomyces cerevisiae. *Genetics* **157**, 1469–1480 (2001).
12. H. Wang, L. B. Carey, Y. Cai, H. Wijnen, B. Futcher, Recruitment of Cln3 cyclin to promoters controls cell cycle entry via histone deacetylase and other targets. *PLoS Biol.* **7**, e1000189 (2009).
13. P. Jorgensen, J. L. Nishikawa, B.-J. Breitkreutz, M. Tyers, Systematic Identification of Pathways That Couple Cell Growth and Division in Yeast. *Science* **297**, 395–400 (2002).
14. S. Schrott, C. Osman, Two mitochondrial HMG-box proteins, Cim1 and Abf2, antagonistically regulate mtDNA copy number in Saccharomyces cerevisiae. *Nucleic Acids Res.* **51**, 11813–11835 (2023).
